# Structural Insights into the Quinolone Resistance Mechanism of *Mycobacterium tuberculosis* DNA Gyrase

**DOI:** 10.1371/journal.pone.0012245

**Published:** 2010-08-18

**Authors:** Jérémie Piton, Stéphanie Petrella, Marc Delarue, Gwénaëlle André-Leroux, Vincent Jarlier, Alexandra Aubry, Claudine Mayer

**Affiliations:** 1 Unité de Dynamique Structurale des Macromolécules, Département de Biologie Structurale et Chimie, Institut Pasteur, Paris, France; 2 URA 2185, CNRS, Paris, France; 3 UPMC Univ Paris 06, Paris, France; 4 UPMC Univ Paris 06, EA1541, Bactériologie-Hygiène, Paris, France; 5 Unité de Biochimie Structurale, Département de Biologie Structurale et Chimie, Institut Pasteur, Paris, France; 6 Université Paris Diderot Paris 7, Paris, France; University of Cambridge, United Kingdom

## Abstract

*Mycobacterium tuberculosis* DNA gyrase, an indispensable nanomachine involved in the regulation of DNA topology, is the only type II topoisomerase present in this organism and is hence the sole target for quinolone action, a crucial drug active against multidrug-resistant tuberculosis. To understand at an atomic level the quinolone resistance mechanism, which emerges in extensively drug resistant tuberculosis, we performed combined functional, biophysical and structural studies of the two individual domains constituting the catalytic DNA gyrase reaction core, namely the Toprim and the breakage-reunion domains. This allowed us to produce a model of the catalytic reaction core in complex with DNA and a quinolone molecule, identifying original mechanistic properties of quinolone binding and clarifying the relationships between amino acid mutations and resistance phenotype of *M. tuberculosis* DNA gyrase. These results are compatible with our previous studies on quinolone resistance. Interestingly, the structure of the entire breakage-reunion domain revealed a new interaction, in which the Quinolone-Binding Pocket (QBP) is blocked by the N-terminal helix of a symmetry-related molecule. This interaction provides useful starting points for designing peptide based inhibitors that target DNA gyrase to prevent its binding to DNA.

## Introduction

Type II topoisomerases are essential and ubiquitous nucleic acid-dependent nanomachines involved in the regulation of DNA topology and especially in the regulation of DNA supercoiling [1]. Type II topoisomerases act by an ATP-dependant double-stranded DNA break [1]. Except archaeal topoisomerase VI [2], [3], they all belong to a single protein superfamily, the type IIA topoisomerases, sharing homologous sequences and overall structures [4]. However, they have acquired distinct functions during evolution [1]. Bacterial genomes usually encode two type IIA enzymes, DNA gyrase and topoisomerase IV. DNA gyrase facilitates DNA unwinding at replication forks and topoisomerase IV has a specialized function in mediating the decatenation of interlocked daughter chromosomes [5]. *Mycobacterium tuberculosis*, the aetiologic agent of tuberculosis, is unusual in possessing only one type II topoisomerase, DNA gyrase [6]. Consequently, the *M. tuberculosis* DNA gyrase exhibits a different activity spectrum as compared to other DNA gyrases, namely it supercoils DNA with an efficiency comparable to that of other DNA gyrases but shows enhanced relaxation, DNA cleavage, and decatenation activities [7].

**Figure 1:**
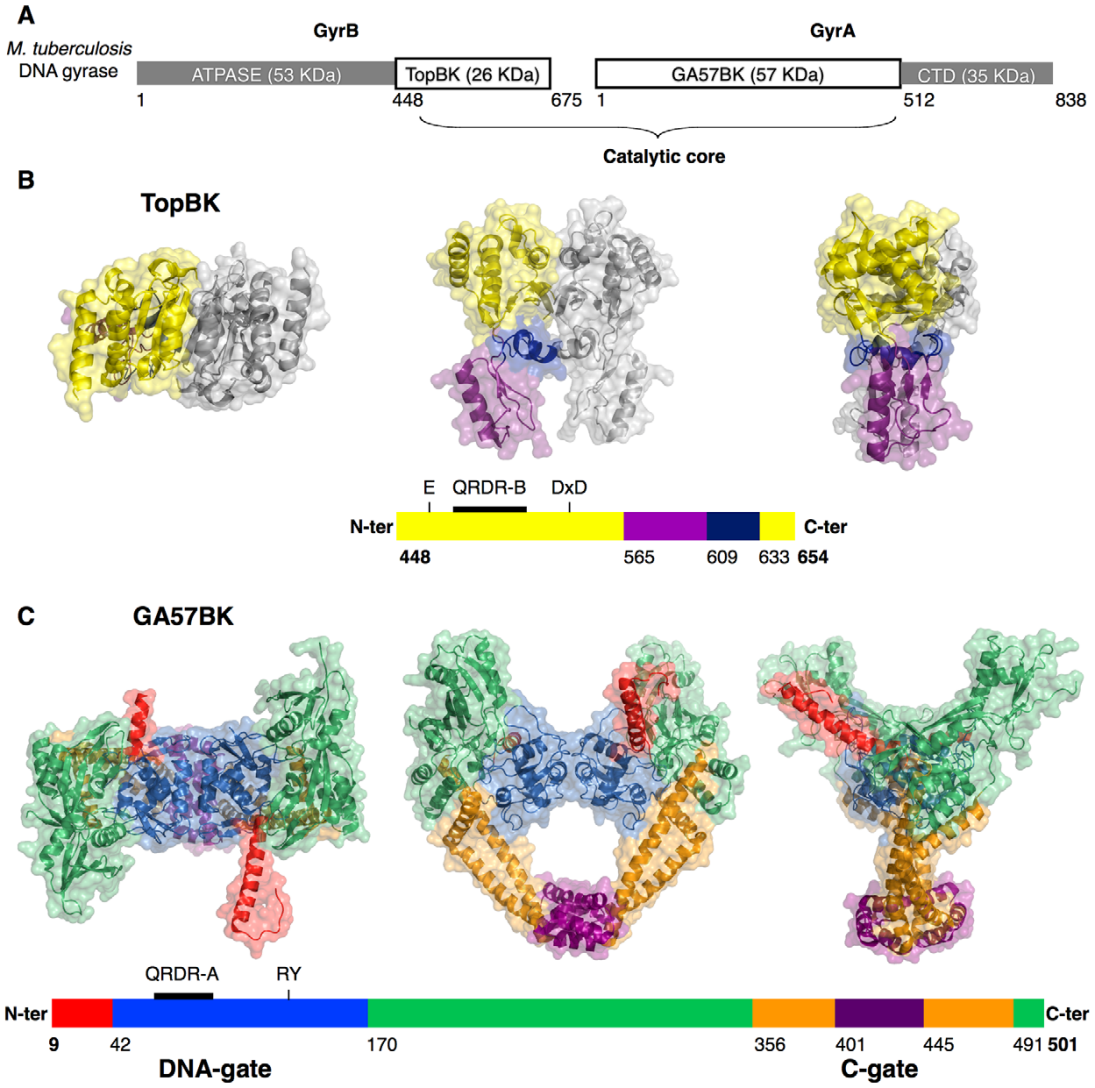
Domain organization and structures of the individual domains from the *M. tuberculosis* DNA gyrase catalytic core. **A.** Domain organization of the *M. tuberculosis* DNA gyrase. The catalytic core is composed by the Toprim domain and the breakage-reunion domain. **B.** Three orthogonal views of the dimeric Toprim domain from *M. tuberculosis* colored by regions. The crystal structure of the complete Toprim domain (TopBK) encompasses residues T448 to E654. The schematically represented primary sequence is colored as in the structure. The N-terminal residue numbers of the regions (Toprim, tail and hinge) and the TopBK C-terminal residue number are indicated. The Toprim region, constituted by discontinuous N- and C-terminal sequence segments and containing the magnesium-binding site (E459, D532 and D534) and the QRDR-B (Quinolone Resistance Determining Region in GyrB) is colored in yellow, the Tail region in purple and the hinge between the two regions in blue. The second monomer generated by a crystallographic two-fold axis is represented in grey. **C.** Three views of the dimeric breakage-reunion domain from *M. tuberculosis* colored by regions. The crystal structure of the complete breakage-reunion domain (GA57BK) extends from D9 to A501. The N-terminal helix is colored in red, the DNA-gate containing the catalytic residues R128 and Y129 and the QRDR-A in blue, the ‘tower’ in green, the helix-bundle in orange and the C-gate in purple.

DNA gyrase and topoisomerase IV consist of two subunits (GyrA and GyrB in DNA gyrase, ParC and ParE in topoisomerase IV), which form the catalytically active heterotetrameric complex (i.e. A_2_B_2_ and C_2_E_2_, respectively). Subunit A consists of two domains, the N-terminal breakage-reunion domain and a carboxy-terminal domain, termed CTD. Subunit B consists of the ATPase domain followed by the Toprim domain. The GyrB Toprim and GyrA breakage-reunion domains come from separate subunits and cooperatively form the enzyme core (Figure 1A). The breakage-reunion domain contains the catalytic tyrosine responsible for the cleavage and religation of the DNA double helix. Although the structure of a fully intact, active type IIA topoisomerase has yet to be determined, structural and biochemical studies of the individual fragments have led several authors to propose a model of its global quaternary structure and a catalytic mechanism of the holoenzyme [8]. The breakage-reunion domain binds a DNA segment termed the ‘gate’ or G-segment at the DNA-gate. The N-terminal ATPase domains dimerize upon ATP binding, capturing the DNA duplex to be transported (T-segment). The T-segment is then passed through a transient break in the G-segment opened by the breakage-reunion domains, the DNA is resealed and the T-segment released through a protein gate, the C-gate, prior to resetting of the enzyme to the open clamp form.

Quinolones, which target the two bacterial type II topoisomerases, exert their powerful antibacterial activity by interfering with the enzymatic reaction cycle. Specifically, they bind to the enzyme-DNA binary complex, thereby stabilizing the covalent enzyme tyrosyl-DNA phosphate ester. The resulting ternary complexes block DNA replication and lead to cell death [9]. Quinolones are one of the most effective second-line drugs in the treatment of multidrug-resistant tuberculosis (MDR-TB; strains resistant to the two main antituberculous drugs, rifampicin and isoniazid) [10] and are currently under study for shortening treatment duration of drug-susceptible tuberculosis [11]. Tuberculosis still remains the leading cause of death from a curable infectious disease causing millions of deaths annually (http://www.who.int). Unfortunately, due to the long and complex nature of TB treatment, inappropriate use of first line antituberculous drugs is common, leading to the emergence of drug-resistant bacilli, especially MDR strains. Widespread dissemination of these bacilli poses a serious threat to global TB control [12]. Compared with *E. coli*, the “intrinsic resistance” of *M. tuberculosis* to quinolones is relatively high, mainly due to the primary structure of DNA gyrase. Namely, amino acids at positions 81 and 90 in GyrA and 482 in GyrB have been demonstrated to be involved in “intrinsic quinolone resistance” [13]. Nonetheless, quinolones, and in particular fluoroquinolones, are essential antibiotics for MDR-TB [13], [14]. However, *M. tuberculosis* develops “acquired resistance” to quinolones following prolonged exposure, leading to the emergence of extensively drug-resistant (XDR) strains (MDR-TB strains resistant to any fluoroquinolone and to at least one of three injectable second-line anti-TB drugs) [15], [16], [17]. This “acquired resistance” is mainly a result of mutations in the DNA gyrase sequence [18], [19]. Mutations conferring bacterial resistance to quinolones occur in two short discrete segments termed the quinolone resistance-determining regions (QRDR) [20] located in the breakage-reunion domain of GyrA subunit (QRDR-A) and less frequently in the Toprim domain of GyrB (QRDR-B) [20], [21], [22]. Among the described mutations, we have unequivocally demonstrated that the nature of the amino acids at positions 88, 90 and 94 in GyrA plays a crucial role in the “acquired resistance” to quinolones (Table 1) [21], [23].

**Table 1:** Mutations described in *M. tuberculosis* strains implicated in “acquired” resistance to quinolones.

Mutation		Effect on quinolone susceptibility	Reference
GyrA	GyrB		
G88A		resistance	13
A90V		resistance	21
D94A, G, N		resistance	21
	N499D	resistance	21
T80A		no effect	21
T80A+A90G		hypersusceptibility	21

Summary of mutations described in *M. tuberculosis* strains (e.g. clinical strains or strains cultured *in vitro* in presence of quinolone in order to select a resistant strain), which have been unequivocally demonstrated as implicated in “acquired” resistance.

The challenge of better understanding the complex mechanism of quinolone resistance in *M. tuberculosis* requires high-resolution structures of the antibiotic targets. Following our previous results, the aim of this work was to obtain a 3-dimensional understanding of the relationships between a given amino acid mutation and quinolone resistance phenotype in *M. tuberculosis*. Simultaneously to our results, two structures of *M. tuberculosis* DNA gyrase domains were published last year, the low resolution GyrB' structure (PDB code 2ZJT, [24]) and the truncated *Mt*GyrA59 domain (PDB code 3ILW, [25]). The first picture of the enzyme-quinolone interactions was given by the low resolution structures of *Streptococcus pneumoniae* ParC breakage-reunion and ParE Toprim domain in complex with DNA and quinolones (PDB codes 3FOF and 3K9F, [26]). Moreover, other efforts to develop new potent catalytic inhibitors of bacterial DNA gyrase were illustrated by the crystal structure of *E. coli* DNA gyrase in complex with the bifunctional antibiotic simocyclinone D8 [27]. Its mode of action is unique in that it directly interacts with DNA gyrase to prevent its binding to DNA.

In this work, we combined X-ray crystallographic studies, sedimentation velocity experiments and activity assays of the two domains that form the enzyme core of *M. tuberculosis* DNA gyrase, the GyrB Toprim and GyrA breakage-reunion domains. We solved two high resolution structures of the Toprim domain displaying two different conformations of the metal-binding site, to 2.1 and 1.95 Å resolution, respectively. The crystal structure of the breakage-reunion domain we solved to 2.7 Å resolution, revealed a promising interaction that will be further exploited for drug design. This interaction involves the N-terminal helix, which is anchored in the active site of a symmetry-related molecule. Additionally, using the crystal structures of both domains, we modeled the catalytic reaction core in complex with DNA and a quinolone. This study brings the first structural explanation on quinolone resistance mechanism of *M. tuberculosis* DNA gyrase.

## Results

### Crystal structures of the Toprim and breakage-reunion domains are biologically relevant

**Figure 2:**
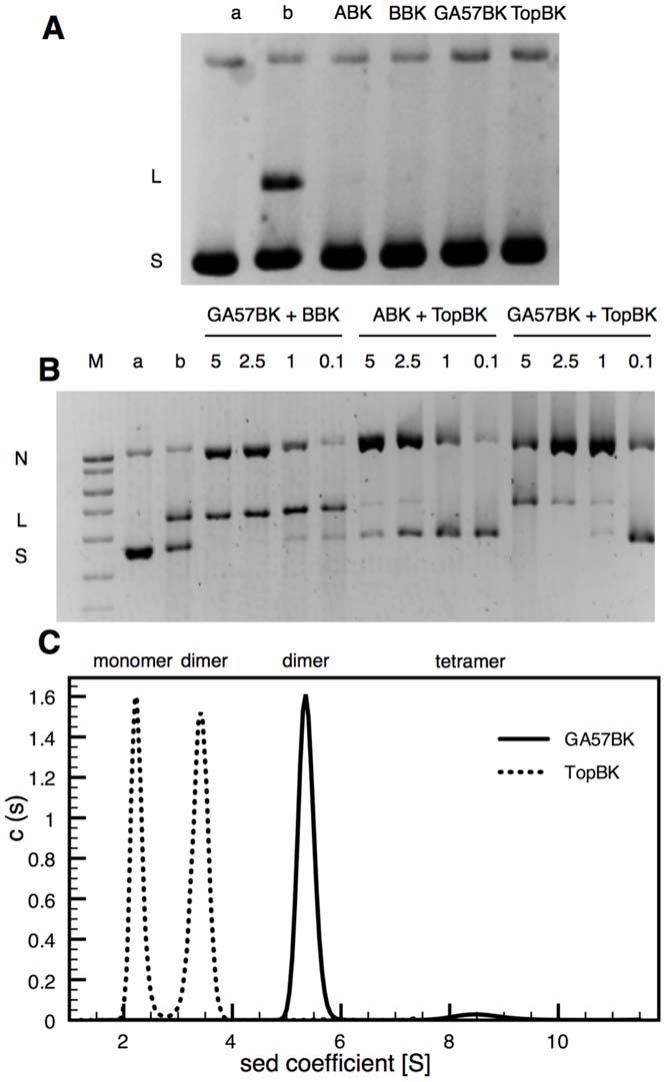
Activity assays and oligomerization of the TopBK and GA57BK domains. **A.** The quinolone-mediated DNA cleavage activity test measured on supercoiled pBR322 DNA (0.4 µg) as a substrate in the presence of moxifloxacin (50 µg/ml) and 2.5 µg of each subunit alone: full length subunit A (ABK), full length subunit B (BBK), GA57BK and TopBK. Lanes a and b are supercoiled pBR322 DNA and control of cleavage activity with WT *M. tuberculosis* DNA gyrase (ABK and BBK), respectively. **B.** The quinolone-mediated DNA cleavage activity test measured on supercoiled pBR322 DNA (0.4 µg) as a substrate in the presence of moxifloxacin (50 µg/ml) with various amounts (indicated by values in µg) of GA57BK associated with the full length subunit B (BBK, 1 µg), various amounts of TopBK with the full length subunit A (ABK, 1 µg), and various amounts (indicated by values in µg) of the binary complex constituted by GA57BK and TopBK. Lanes M, a and b are DNA size markers, supercoiled pBR322 DNA and control of cleavage activity with WT *M. tuberculosis* DNA gyrase (ABK and BBK), respectively. N, L and S denote nicked, linear and supercoiled DNA, respectively. **C.** Sedimentation experiments of GA57BK and TopBK. The single peak of GA57BK corresponds to the dimer, with a sedimentation coefficient of 5.4±0.2 S. The two peaks observed for TopBK correspond to the monomeric and dimeric form, with sedimentation coefficients of 2.3±0.1 S and 3.4±0.2 S, respectively. c(s) on the y-axis designates the distribution of the sedimentation coefficients observed for the experiment.

The C-terminal GyrB domain (Toprim domain, residues 448–654) and the entire N-terminal GyrA domain (breakage-reunion domain, known as GyrA59 in *E. coli*, residues 1–502), hereafter named TopBK and GA57BK, respectively, were overproduced and purified. DNA cleavage activity assays show that TopBK is able to catalyze DNA breaks when associated to the full-length A subunit. Similarly, GA57BK is able to catalyze DNA breaks when associated with the full-length B subunit (Figure 2A and B). Interestingly, the GA57BK-TopBK complex has DNA cleavage activity, showing that these domains possess all determinants for DNA cleavage and confirming that these two domains form the catalytic reaction core of the *M. tuberculosis* DNA gyrase (Figure 2A and B). In addition to DNA cleavage, some nicking is also observed when TopBK is associated either with the full length GyrA, or with GA57BK (Figure 2B). This could be the result of a decrease in the complex stability when TopBK is used in the activity assays.

TopBK was crystallized in presence of magnesium (*crystal I*) and calcium (*crystal II*) and the structures were solved at 2.1 Å and 1.95 Å resolution, respectively, with one monomer in the asymmetric unit in both cases. Slight modifications of the previously described crystallization conditions [24], [28], e.g. modifying the pH value and adding divalent cations, led to a space group change and a substantial increase in the resolution (2.8 to 1.95 Å). A crystallographic two-fold axis generates a dimeric structure, similar to the dimer observed in the asymmetric unit of the GyrB' structure (2ZJT). GA57BK corresponds to the entire N-terminal domain with a molecular mass of 57 kDa. The crystals belong to space group C2, with a dimer in the asymmetric unit. Clear electron density was observed for the N-terminal fragment that could be built either entirely (chain A) or partially (chain B) because of different crystal contacts. Consequently, the final model spans residues 9 to 499 for chain A and 29 to 501 for chain B.

Both TopBK and GA57BK display a dimeric structure in the crystal (Figure 1B and C). The biological relevance of these dimeric forms was investigated using analytical ultracentrifugation. Sedimentation experiments reveal that TopBK and GA57BK exhibit different behaviour in solution. In the case of TopBK, two species are observed with a 50/50 distribution when using a protein concentration corresponding to the crystallization conditions (Figure 2C). The two species display a sedimentation coefficient of 2.3±0.1 S and 3.6±0.2 S, corresponding to the monomer and the dimer, respectively, according to the theoretical sedimentation coefficient values calculated from the crystallographic structure (2.2 and 3.5 S, respectively). In contrast, GA57BK is mainly dimeric in solution (Figure 2C). Sedimentation experiments showed that one species was observed with a sedimentation coefficient of 5.4±0.2 S, compatible with the value calculated from the crystallographic dimer structure (5.6 S). The good agreement between these experimental and theoretical values indicates that the dimeric conformation of GA57BK is stable in solution. These results suggest that the biological unit is a dimer.

### The crystal structure of the isolated Toprim domain is a dimer

**Figure 3:**
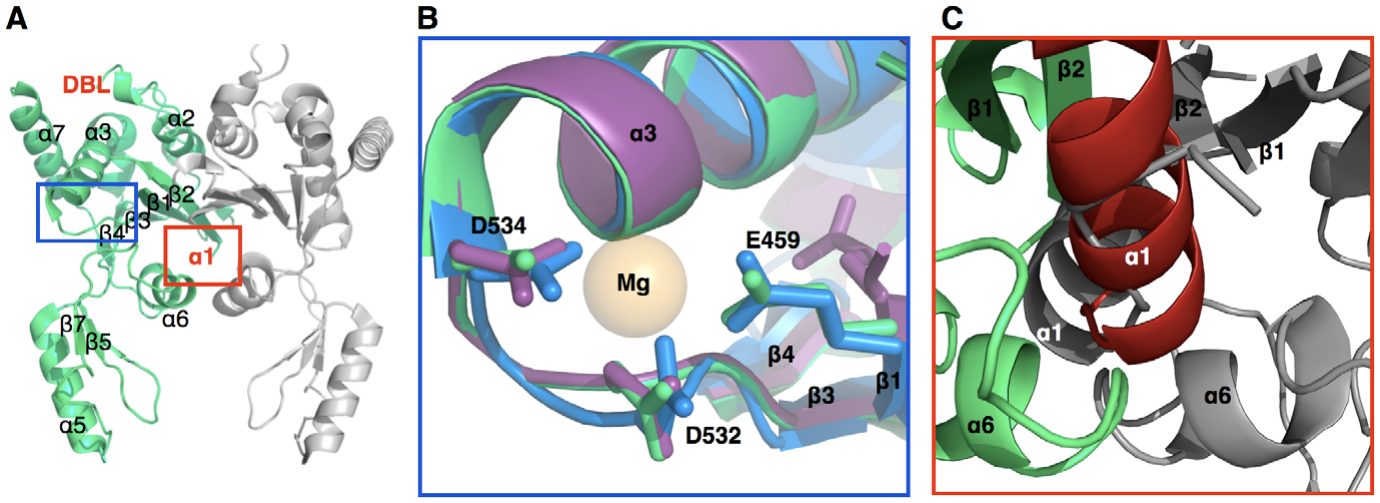
The TopBK magnesium-binding site. **A.** Overall view of the dimeric structure of the Toprim domain from *M. tuberculosis*. One monomer constituting the asymmetric unit is represented in green, the second monomer generated by a crystallographic two-fold axis in grey. The secondary structures are indicated by black labels. The locations of the two disordered regions, the DNA Binding Loop (DBL) and the α1-helix, are indicated by the red labels “DBL” and “α1”, respectively. **B.** The magnesium-binding site of both *M. tuberculosis* TopBK structures, TopBK *crystal I* (3IFZ, in green) and TopBK *crystal II* (3M4I, in purple) with the conserved residues, E459, D532 and D534. The active site of the *S. cerevisiae* Toprim domain (2RGR) is represented in blue and its bound magnesium ion in orange. **C.** Close view of the TopBK dimer interface. The two symmetry-related α1 helices (shown in red and grey) generate steric clashes.

The overall fold of the TopBK structure is very similar in both crystal forms, and also similar to the previously published GyrB' structure (2ZJT) [24] and to the Toprim domain of the known eukaryotic counterpart, the yeast topoisomerase II [29]. The structure displays a two-domain organization, a globular domain constituted by discontinuous segments (residues 448–564 and 633–654) and the Tail domain (residues 565–608) connected by a loop-helix-loop hinge region (residues 609–632) (Figure 1B). The globular domain, organized in a Rossmann-like fold, contains the Toprim domain described by Aravind and collaborators [30] and the QRDR-B (residues 461–499) (Figure 3A and S1). The Tail domain comprises a three-stranded antiparallel β-sheet and an α-helix. In the globular domain, the conserved acidic triad (E459, D532, D534), which constitutes the signature of the Toprim domain, binds the magnesium ion essential for the catalysis of the cleavage-ligation reaction. In the structure of *crystal I*, the magnesium ion is not visible, despite being present in the crystallization condition. However, side chains of the catalytic triad are in conformations which would allow ion coordination, as observed in the *yeast* topoisomerase II in complex with DNA (Figure 3B). Presumably, the ion is not bound due to the absence of the DNA. When magnesium is substituted in the crystallization conditions by calcium (TopBK *crystal II*), side chains of the triad are observed in an inactive conformation similar to the one observed for the low resolution *M. tuberculosis* Toprim domain structure [24].

The Toprim domain forms a dimer with a symmetry related molecule in both crystal structures (*crystal I* and *II*), burying 1017 Å^2^ at the protein-protein interface, indicative of a biologically relevant interaction. The two species observed in sedimentation experiments with a 50/50 distribution are identified as the monomeric TopBK domain and the crystallographic dimer suggesting that this crystallographic dimer exists in solution outside the context of the full-length subunit.

Surprisingly, the high resolution structures of TopBK, revealed two disordered regions, between β1 and β2 (residues 460–474) and between β2 and α2 (residues 484–492) (Figure 3A). These regions are structured in the context of the catalytic core or in presence of DNA. The first disordered region corresponds to the α1-helix [30], as observed in the three structures of the yeast topoisomerase II [29], [31], [32] and in the structure of the *S. pneumoniae* reaction core [26]. Interestingly, this region is located at the dimer interface and placing an α-helix would generate steric hindrance between the two helices of the crystallographic related monomers (Figure 3C). The second disordered region, the loop between β2 and α2, is exposed to the solvent explaining its high flexibility. In the structures of type II topoisomerases in complex with DNA, this loop (hereafter named DBL for DNA-Binding Loop) constitutes the interface between the Toprim domain and DNA and is stabilized through protein-DNA interactions.

### The breakage-reunion domain is in a closed conformation

GA57BK forms a biological dimer in the asymmetric unit, generating a heart-like shaped structure with outer dimensions of 100×100×90 Å (Figure 1C) and a central hole of 30 Å diameter allows the passage of the T-segment from the DNA-gate to the C-gate. GA57BK forms a biological dimer in a ‘closed’ conformation in the asymmetric unit, as the C-gate, which constitutes the so-called primary dimer interface, and the DNA-gate, the secondary protein-protein interface, are both closed (Figure S2). This closed conformation is observed in all isolated breakage-reunion domain structures, the *Mt*GyrA59 from *M. tuberculosis* [25], GyrA59 from *E. coli* and of the two topoisomerase IV structures from *S. pneumoniae* [33] and from *S. aureus* [34]. This shows that the closed conformation is stable and energetically favorable. This stability is essential to generate the interface needed to trap the DNA G-fragment in order to start the topoisomerase cycle. This is in agreement with FRET experiments showing that the DNA-gate of the *Bacillus subtilis* DNA gyrase is predominantly in the closed conformation during the DNA relaxation and supercoiling reactions [35]. When comparing all five protein-protein interfaces (sum of DNA- and C-gate interfaces), the highest value is observed for both structures of *M. tuberculosis* DNA gyrase (Table S1). Whereas the C-gate displays similar values, ranging from 1029 to 1120 Å^2^, differences in interface area are observed at the DNA-gate with a value of more than 800 Å^2^ for *M. tuberculosis*, representing nearly one half of the total interface. Both structures confirm that the *M. tuberculosis* breakage-reunion domain has a compact closed conformation, especially at the level of the DNA-gate, whatever the crystal environment.

Each monomer of GA57BK contains five distinct regions, the N-terminal fragment (residues 9–41), deleted in *Mt*GyrA59 and disordered in the homologous structures, and the four typically observed regions in breakage-reunion domains of all type II topoisomerases (Figure 1C and S3). In this way, GA57BK resembles the type II topoisomerase structures in complex with Toprim, namely the yeast topoisomerase II or the structure of the complex between ParC, ParE, DNA and a fluoroquinolone (see below). The next four domains, the DNA-gate (residues 42–169), the ‘tower’ (residues 170–355 and 491–501), the C-gate (residues 401–444) and the three-helix bundle (residues 356–400 and 445–490) (Figure 1C), exhibit an overall structural fold similar to that observed for other bacterial type II topoisomerases [33], [34], [36] and the yeast topoisomerase II [29], [31], [32]. The DNA-binding helix-turn-helix motif (α3 and α4 helices), the QRDR-A (residues 74–113) and the catalytic residues involved in DNA cleavage, namely R128 and Y129, are localised in the DNA-gate.

### The active site is blocked through crystal contacts established by the N-terminal helix

**Figure 4:**
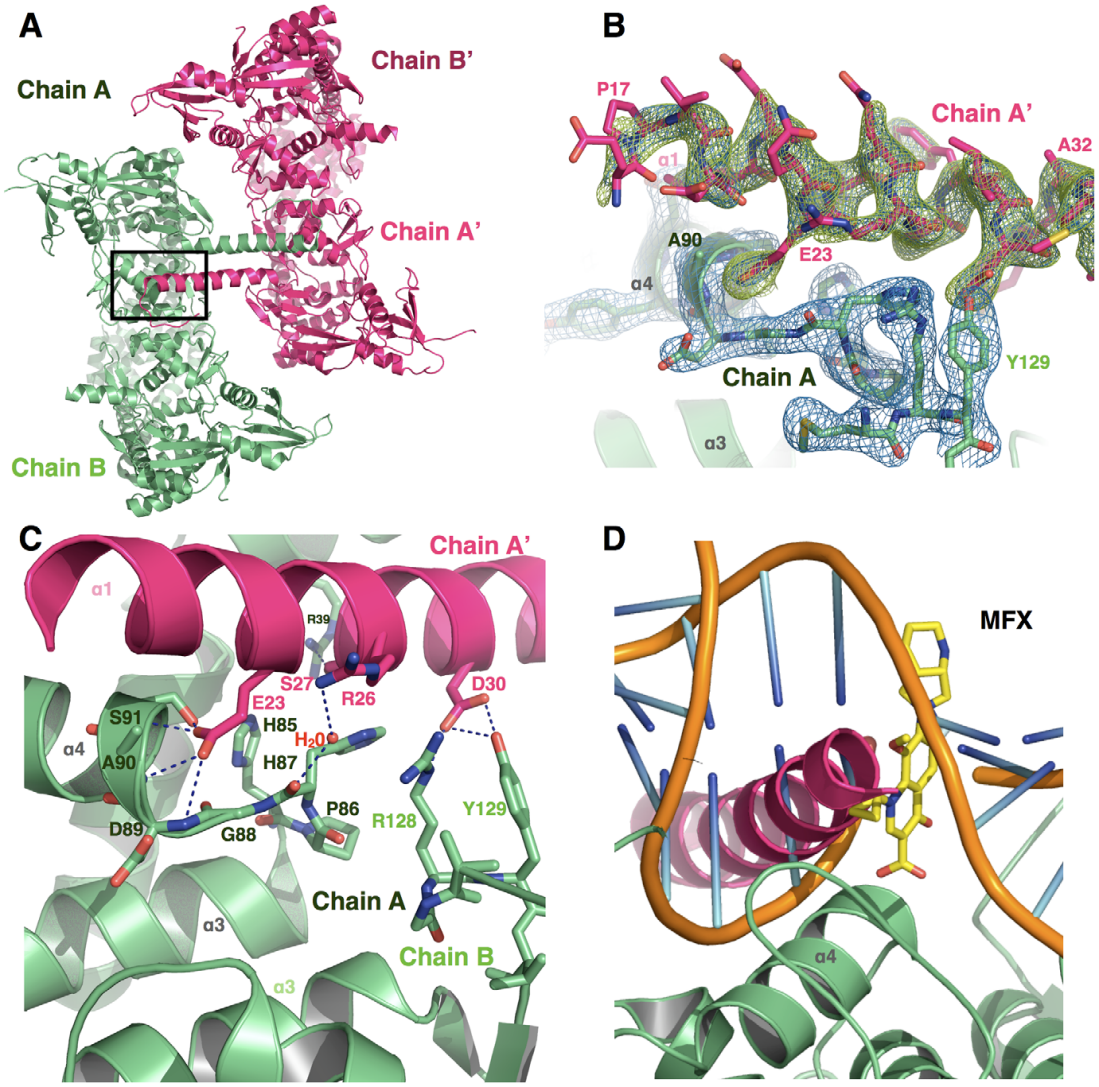
The active site of *M. tuberculosis* DNA gyrase is blocked by the N-terminal helix of a symmetry-related molecule. **A.** Two dimers of GA57BK, related by the crystallographic two-fold axis, interact through the N-terminal helix. **B.** Omit maps for the N-terminal helix. The (2F_obs_ – F_calc_) map shown in blue is contoured at 1.5 σ whilst the (F_obs_ – F_calc_) map shown in green is contoured at 3 σ. **C.** Detailed interactions of the N-terminal helix (chain A', in hot pink) in the active site of the symmetry-related molecule (chain A, in light green). Y31 of the N-terminal helix and R54 of the symmetry-related molecule are located on the back-side of the helix and are not represented for better clarity. **D.** Based on the model discussed in the text, the N-terminal helix (chain A', in hot pink) occupies the quinolone-binding pocket (QBP) and clashes with the modeled DNA, represented in orange, and the fluoroquinolone, in yellow, bound to the QBP.

In contrast to other structures of the breakage-reunion domain alone (*i.e. E. coli* DNA gyrase, *S. aureus* and *S. pneumoniae* topoisomerase IV), the N-terminal segment of GA57BK (residues 9–41) is ordered and is organized in two distinct secondary structures (Figure 4A). Residues D9 to E16 form a loop whose B factors indicate high flexibility, followed by a 24-residue long α-helix (Figure 4B). Until now, this helix was only observed when the Toprim domain is also present, whether DNA is complexed (in the structure of the *S. pneumoniae* topoisomerase IV catalytic core in complex with DNA, 3FOF [26] and the yeast topoisomerase II catalytic core-DNA complex, 2RGR [29]) or not (in the two structures of the yeast topoisomerase II catalytic core, 1BJT [32] and 1BGW [31]).

A previously unobserved feature of our crystal structure of GA57BK is the interaction between this N-terminal region with neighbouring molecules in the crystal packing. As shown in Figure 4, the N-terminal fragment residues of chain A in a given asymmetric unit clearly establish direct contacts with the active site residues of its nearest neighbour (chain A') in the adjacent asymmetric unit. As these two molecules are related by the crystallographic two-fold axis, this interaction is reciprocal. The α-helix is deeply anchored in the active site of its neighbouring molecule. Several hydrogen-bonding interactions link E23 from the α-helix to the α3–α4 region, namely D89, A90 and S91 from the symmetry-related molecule (Figure 4C). R26 links the main chain carbonyl-group of H87 *via* a water molecule. In addition, D30 establishes hydrogen bonds with the hydroxyl group of the catalytic tyrosine (Y129) and a salt bridge with the catalytic arginine (R128). Finally, on the opposite face of the N-terminal helix, S27 and Y31 form an H-bonding network with R39 and R54 from the symmetry-related molecule (Figure 4C). This arrangement buries a surface area of 1227 Å^2^, indicating a stable interaction. The resulting tetramer could explain the small peak observed in sedimentation experiments (Figure 2B). Further studies exploiting this interaction for drug design will be investigated. A peptide of 16 amino acids corresponding to residues 15 to 30 of the *M. tuberculosis* breakage-reunion domain will be used as an inhibitor for *M. tuberculosis* DNA gyrase in activity and binding assays in order to develop structure-activity relationships. Combined docking and molecular dynamics simulations will be used to design small molecules that mimic the peptide-active site interactions [37].

### The *M. tuberculosis* breakage-reunion domain possesses two specific structural motifs

**Figure 5:**
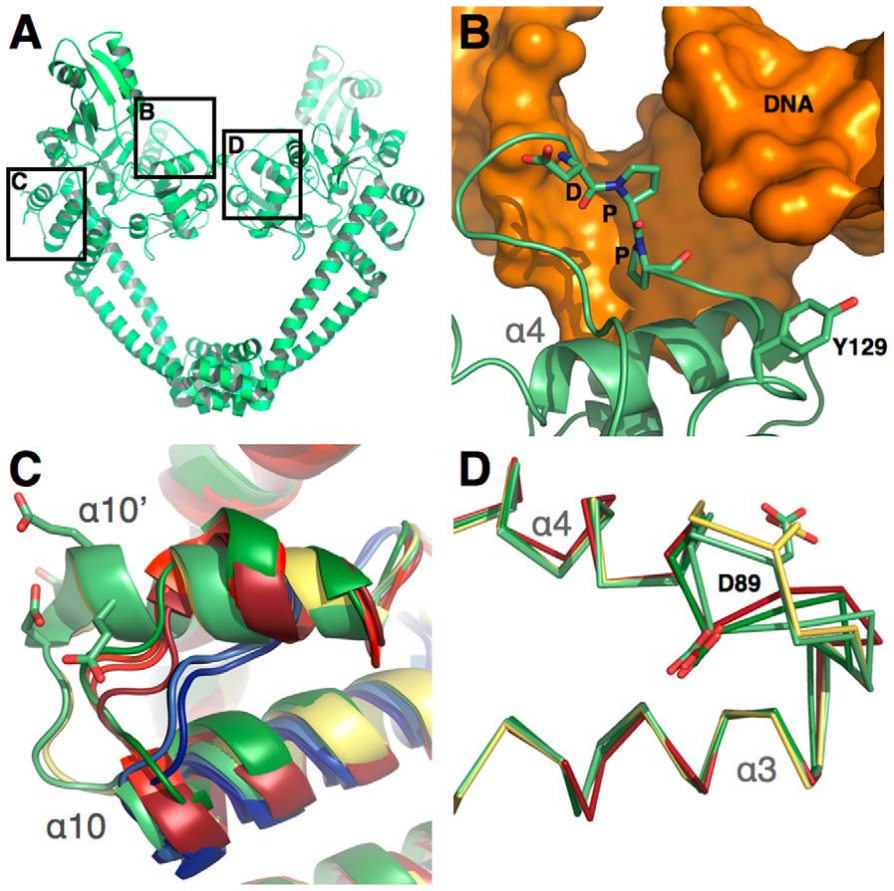
Comparison of the *M. tuberculosis* breakage-reunion domain to other type II topoisomerase structures. **A.** Global view of the breakage-reunion domain. The boxes indicate the three close-up views shown in B, C and D. **B.** The DPP loop of GA57BK represented in light green is near the DNA phosphate backbone, in orange (see text for details of the model). **C.** Close view of the α10–α10' loop. Both *M. tuberculosis* structures, GA57BK (represented in light green) and *Mt*GyrA59 (in yellow) possess a DEEX sequence insertion in this loop. The conformation of this loop is different in other bacterial type II topoisomerases, namely the three topoisomerase IV structures represented in red and *E. coli* GyrA59 in green, and in the three yeast topoisomerase II structures in blue. **D.** Close-up view of the α3–α4 loop. The conformations of GA57BK chain B (light green), and *Mt*GyrA59 (yellow) are different from the conformation of GA57BK chain A (light green) and *E. coli* GyrA59 (dark green).

Unexpectedly, structural comparison of *M. tuberculosis* DNA gyrase to other type II topoisomerases clearly reveals that there is no significant difference between a DNA gyrase from species containing only one type II topoisomerase and the other type II topoisomerases, DNA gyrase and topoisomerase IV, generally found in bacteria (Figure S4). However, we found that two regions could be correlated to the wider substrate spectrum of *M. tuberculosis* DNA gyrase function. First, a sequence motif (DPP) in the loop between the α3–α4 DNA-binding motif and the catalytic tyrosine residue resembles the sequence observed in topoisomerases IV and is rarely observed in DNA gyrase sequences. Localised at the side of the DNA-gate and in direct interaction with DNA (Figure 5A and B), this loop could contribute to the topoisomerase IV-like activity (i.e. decatenation) of *M. tuberculosis* DNA gyrase. Second, a specific insertion in the *M. tuberculosis* sequence consists in a negatively charged motif DEEE (residues 211–214) (Figure S3). In the structure, this motif is localised at the solvent-exposed surface of the tower domain in the α10-loop-α10' region (Figure 5C). SAXS studies showed that this region interacts with the GyrA CTD [38]. Superimposition of the different breakage-reunion domains shows that the structures display significant differences in this region and can be clustered in three distinct groups according to the conformation of the loop (Figure 5C). First, the eukaryotic topoisomerase II group, represented by the three different structures of the *S. cerevisiae* topoisomerase II, is characterized by the absence of the helices α10'. The second group, which contains the bacterial type IIA topoisomerases (topoisomerase IV or DNA gyrase) from organisms containing two topoisomerases, possess a short α10'. The interaction between this region and the CTD might therefore be different in these two groups suggesting that this region may be implicated in functional specificity of type II topoisomerases, as the CTD plays a crucial role in DNA interaction. Finally, the two structures of *M. tuberculosis* constitute the third group. The DEEE motif creates an extension of the α10' helix modifying the CTD interface and could thus play an important role during the catalytic cycle of the *M. tuberculosis* DNA gyrase. To confirm the relationships between these two specific structural motifs and the function of *M. tuberculosis* DNA gyrase, the role of the DPP and the DEEE motifs will be studied through site-directed mutagenesis.

### Structural modeling of the catalytic reaction core in complex with DNA and quinolone

**Figure 6:**
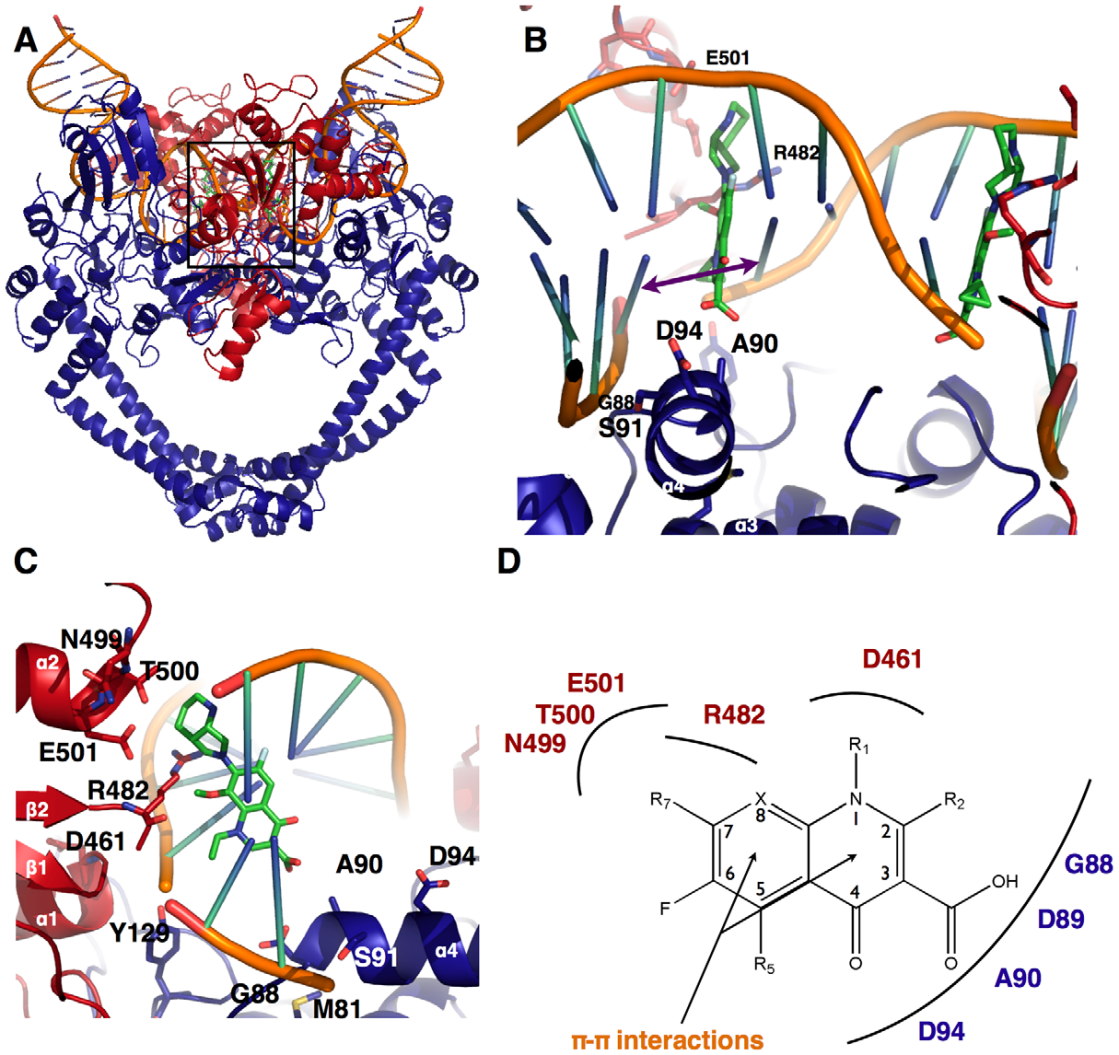
Model of the catalytic reaction core in complex with DNA and moxifloxacin. **A.** Overall structure of the complex. GA57BK is represented in blue, TopBK in red, the DNA in orange and the moxifloxacin in green. **B.** Close-up view of the two quinolone-binding pockets (QBP). The purple arrow highlights the rise of the intercalated base step that constitutes the DNA walls of the QBP. Protein residues that constitute the QBP protein walls are indicated in red for TopBk and blue for GA57BK. The residues shown in sticks belong to the QRDR and are implicated in quinolone resistance. **C.** Close-up view along the DNA axis of one of the two QBP. The same residues as in B are represented in sticks. **D.** Schematic representation of the interactions between QBP residues and chemical groups of the quinolone.

During the catalytic cycle of DNA gyrase, a ternary complex is formed between the Toprim and the breakage-reunion domains and DNA. Quinolones target this complex and inhibit the enzyme through stabilization of the covalent DNA-protein complex formed during catalysis. To explore the mechanistic implications of the *M. tuberculosis* DNA gyrase and to understand how the N-terminal helix would interfere in the context of the complex structure, we performed structural modeling of the cleavage complex based on the structure of a topoisomerase IV complex [26]. This quaternary complex is composed of the catalytic reaction core consisting of the breakage-reunion domain (GA57BK), the Toprim domain (TopBK), a 34-bp DNA duplex and one of the most promising fourth-generation fluoroquinolone, moxifloxacin. In the structure of the complex, DNA is settled on the DNA gate, is linked covalently to the two catalytic tyrosines 129, and is maintained on each side by the ‘tower’ of the breakage-reunion domain and the Toprim domain (Figure 6). The two catalytic sites related by the heterotetramer two-fold axis are separated by four base-pairs and each catalytic site contains one quinolone molecule (Figure 6A and B). The quinolone carboxylate group points towards the major groove, and the R7 group is localised in the minor groove. The interaction energy between each quinolone molecule and its devoted binding pocket is −105 and −112 kcal/mol, respectively. The slight discrepancy could reflect some sequential binding. However, those values evidence a very good binding affinity that is illustrated in Figures 6B and C.

## Discussion

In the present work, we have structurally characterized the two components of the catalytic reaction core. The structure of the breakage-reunion domain (known as GyrA59 in *E. coli*) reveals a new interaction promising for drug design, whilst the high resolution structures of the Toprim domain highlights two disordered regions that play a crucial role during the catalytic reaction of DNA gyrase. The strong point of this study is that we could identify original mechanistic properties of quinolone binding that clarify relationships between amino acid mutations and resistance phenotype. These structure-mechanism relationships have been established from the modeling of the catalytic reaction core based on the two crystal structures, DNA and quinolone, using the crystal structure of the cleavage complex formed by the *S. pneumoniae* breakage-reunion and Toprim domains of topoisomerase IV stabilized by a fluoroquinolone [26].

### The Quinolone-Binding Pocket (QBP), a drug-binding pocket composed of protein and DNA residues

**Figure 7:**
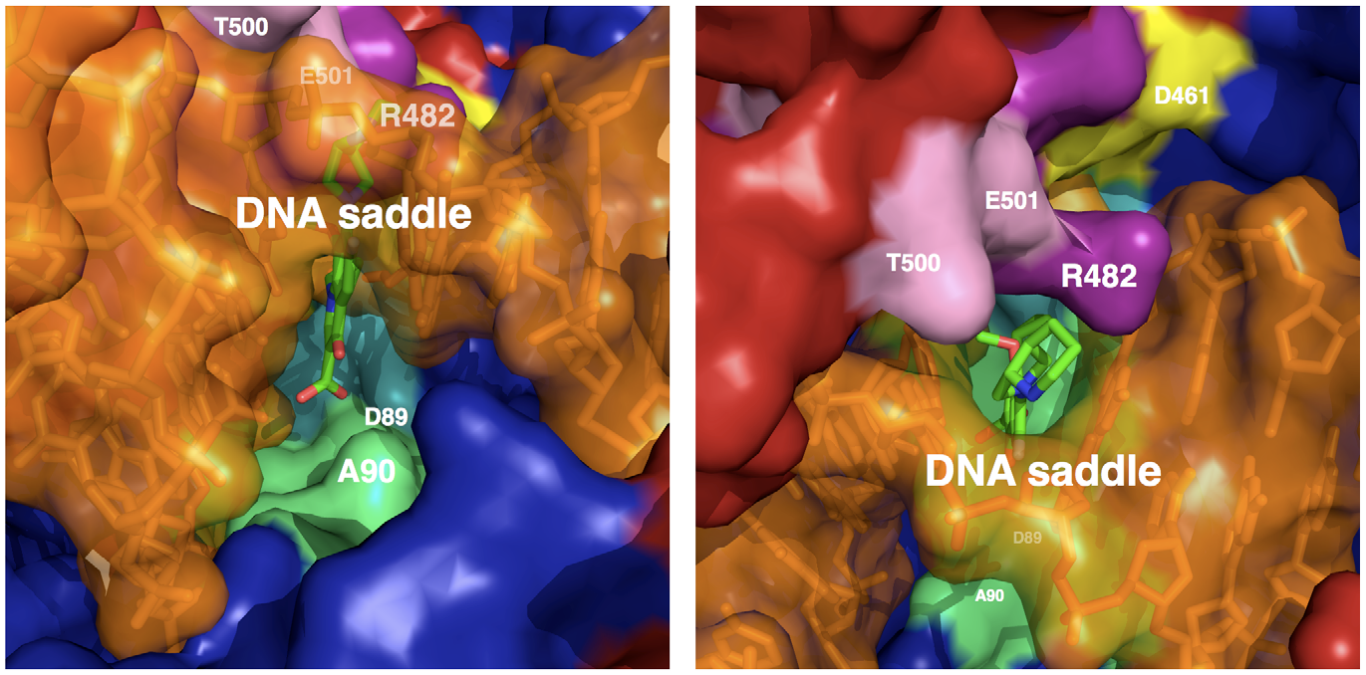
Two views of the Quinolone-Binding Pocket (QBP). The DNA-protein complex is represented in molecular surface and moxifloxacine in sticks. GA57BK is colored in dark blue, TopBK in firebrick, DNA in orange and moxifloxacin in green. The residues of TopBK belonging to the QBP are colored in yellow for the β1-α1 loop residues, in purple for the β2-DBL residues (including R482), and in pink for the DBL-α2 residues. The residues of GA57BK belonging to the QBP are represented in light green and correspond to the α3–α4 region.

Whereas the structures of the *S. pneumoniae* topoisomerase IV and the *M. tuberculosis* DNA gyrase reaction core are very similar, our model allowed us to establish clear relationships between amino acid mutation and resistance phenotype in *M. tuberculosis* DNA gyrase. We propose that the atypical quinolone-binding mode in the Quinolone-Binding Pocket (QBP), whose walls are constituted not only by regions of the Toprim and the breakage-reunion domains but also by DNA (Figure 6B), explains the effect of the amino acid nature at a given position on the observed resistance. The drug is intercalated between the dinucleotide step for which the DNA backbone of one strand is broken (the phosphorus atom is covalently linked to oxygen atom of the catalytic tyrosine). The intercalated dinucleotide step is strongly perturbed, with a twist of nearly 10° and a rise of 7.3 Å (36° and 3.4 Å for a canonical B-helix, 33° and 2.7 Å for A-DNA), typical of an intercalation mechanism (as observed, for example, in the structure of a DNA-nogalamycin complex [39]). The two intercalated base pairs form a saddle, where quinolone is stabilised through π-π interactions (Figure 6B and 7). The quinolone molecule is blocked in this DNA saddle mainly by Van der Waals contacts with residues of both protein domains (Figure 6C, D and 7). On one side, the carboxylate and the R2 groups (R2 is a hydrogen atom in the moxifloxacin) of the drug are maintained by the α3–α4 loop and the beginning of the α4-helix of the breakage-reunion domain (residues 86–91). On the other side, quinolone is immobilized by three regions of the Toprim domain. The β1-α1 loop (residues 459–462) interacts with the R1 group, the β2-DBL loop (residues 480–486) with the R7–R8 group and the beginning of α2 (residues 498–502) with the R7 group (Figure 6C and D). Consequently, both deformation (rise) of the intercalated dinucleotide step forming the DNA saddle, and the specific sequence of the QRDR-A and B, are required to build up the QBP and determine the geometrical characteristics of the binding pocket (volume and shape). In addition, the conformation of the loop connecting helices α3 and α4 (residues 84–88) also affects the depth of the QBP (Figure 6C and D). Whereas this loop displays two different conformations in the two monomers in the GA57BK crystal structure (Figure 5D), our model clearly shows that the presence of DNA tends to push this loop towards the conformation observed in the *E. coli* structure, suggesting that only this conformation is observed when the QBP is formed.

### Structural insights into the mechanism of “intrinsic resistance” to quinolone

The three residues, M81 and A90 in GyrA and R482 in GyrB have been shown to be implicated in “intrinsic” quinolone resistance of *M. tuberculosis* [13], [14]. We have previously demonstrated that A90S and R482K substitutions (S and K are the corresponding residues in *E. coli*) have direct effect on resistance level [13]. In our model, A90 and R482 are part of the QBP, residing on the α4-helix and in the β2-DBL, respectively. As shown in Figure 6C, A90 side chain is oriented toward the carboxylate group of the quinolone. The substitution of this alanine for serine could increase the stability of the drug through a hydrogen bond between the serine side chain and the hydroxyl group of the quinolone, as observed in the *S. pneumoniae* complex (3K9F). Furthermore, the R482 side chain is located in the minor groove and forms a gate which blocks the quinolone in the pocket (Figure 7). It has been shown that removing a lysine from the minor groove energetically costs more than removing an arginine [40]. The gate will open more easily when the residue at this position is an arginine, in contrast to lysine, contributing to the destabilisation of the quinolone in the QBP. This open-close mechanism of the gate could play a role in the “intrinsic resistance” mechanism. Finally, in our previous study, we showed that M81I substitution (I in *E. coli*) alone had not any effect, but could raise the quinolone susceptibility when associated with the A90S mutation [13]. This correlates well with the fact that M81 is not directly located in the QBP. This residue is spatially too far from the quinolone-binding site, but it could affect the QBP by altering the conformation of the α4-helix through direct interactions of the residue at position 81 and two residues of the helix, namely D89 and I92. All these observations show that direct interaction has direct effect on the resistance level, and gives a synergetic effect role to the amino acid nature at position 81. The role of amino acids at position 81 and 90 in “intrinsic resistance” will be further investigated through structural studies of the *M. tuberculosis* DNA gyrase double mutant A90S-M81I in complex with DNA and a quinolone.

### Structural insights into the mechanism of “acquired resistance” to quinolone

A number of mutations that lead to fluoroquinolone “acquired resistance” have been described in the literature [15], [16]. They are all localised in the QRDR-A and -B (residues 74–113 of the GA57BK structure and 461–499 of the TopBK structure, respectively). Interestingly, our model shows that all the residues in the QRDR implicated in the “acquired resistance” are localised in the QBP (as defined above), highlighting the relationships between the QRDR of both subunits and the structurally identified QBP, as previously suggested [41]. The model showed that the overall geometry of the QBP, rather than the network of H-bonding, is crucial for the recognition and binding of quinolone in the pocket. Consequently, amino acid changes in the QBP will lead to modification of the pocket geometry, either (i) directly, for residues whose side chains point into the QBP or, most importantly, (ii) indirectly, through modification of the DNA structure, for residues interacting with the DNA moiety of the QBP. Mutations implicated in nearly ninety percent of the resistant strains are located in the QRDR-A at positions 90 and 94. Interestingly, only A90, which also contributes to the intrinsic resistance of *M. tuberculosis*, interacts through a CH-O bond between its methyl group with the quinolone carboxylate group. Substitution by a valine could generate steric hindrance and this could explain why this mutation is known to increase quinolone resistance [42]. Mutations at other positions on the α4-helix affect the DNA backbone structure by changing the major groove dimensions, as DNA stacks on the α4-helix (Figure 6B and C). Consequently, the size of the saddle formed by the intercalated base pairs will be modified (Figure 7). This size modification could affect the binding and the stability of the drug in the QBP. To illustrate this mechanism, the amino acid at position 94 has a paradoxical effect on the resistance level. Indeed, substitution by either smaller residues like glycine or alanine and bulky residues like tyrosine both increase the resistance level [21]. These residues will either expand or reduce the volume of the pocket, leading to instability of the quinolone in the QBP. Mutations in the QRDR-B, like N499, are much less frequent, but their effects on DNA gyrase activity can also be explained by this shape recognition mechanism. All these observations can be used to improve the efficacy of already existing quinolones.

### Conclusion

Taken together with our previous work concerning the role of specific residues implicated in quinolone resistance [13], [23], our structural results concerning the *M. tuberculosis* breakage-reunion and Toprim domains and the modeled complex of the catalytic reaction core provide key insights into the relationship between the amino acid sequence of the *M. tuberculosis* DNA gyrase and the resistance mechanism to quinolones, a major class of antibiotics against this pathogen. In addition, these results highlight two directions for future work. First, *M. tuberculosis* DNA gyrase, the single type II topoisomerase in this organism, possesses two specific structural motifs, the DEEE loop and the DPP loop, which could partially explain its different activity spectrum as compared to topoisomerase IV or DNA gyrase. Hence, this atypical activity spectrum could be explained by the unique nature of the amino acids present in the DNA gate. Second, the N-terminal helix of the GA57BK structure is structurally ordered and stabilised through crystal contacts. Interestingly, this helix blocks the active site of a symmetry-related molecule through interactions with residues of the α3–α4 loop. In the asymmetric unit, the dimeric structure displays two different conformations for this loop. In agreement with what was proposed by Tretter *et al.* [25], this suggests that this region is conformationally dynamic (Figure 5D). Furthermore, this helix contacts active site residues important for the catalysis of the breakage-ligation reaction. The presence of this N-terminal helix would prevent DNA binding (Figure 4D). These observations will be exploited for the design of a new inhibitor family using peptide-based approaches that target DNA gyrase by competitive inhibition of DNA binding. Thus, they open up new avenues for the development of novel peptide-based DNA gyrase inhibitors, providing valuable new strategies to combat this disease as strains resistant to the current repertoire of drugs are emerging.

## Materials and Methods

### Cloning, expression, purification and crystallization of GA57BK

The breakage-reunion domain of DNA gyrase subunit A from *M. tuberculosis* (residues 1–502), hereafter named GA57BK because of its molecular weight of 57 kDa, was cloned, expressed and purified as reported previously [43]. Briefly, the PCR amplified construct was ligated into the pET-29a vector (Novagen) between the NdeI and XhoI sites. The C-terminal His-tagged protein was overproduced after transforming the plasmid into Rosetta 2(DE3) pLysS (novagen), and purified with a Ni-NTA column and a size exclusion chromatography using Superdex-75 10/300 (GE Healthcare). The protein was concentrated to 10–15 mg/ml in 100 mM Tris-HCl pH 8.

Ga57BK crystals were prepared using the hanging drop vapor diffusion method, mixing 2 volumes of protein sample against 1 volume of reservoir solution [100 mM Sodium HEPES pH 7.5, 4% PEG 4000, 30% MPD]. Crystals grew after several days at 21°C to a maximum size of 200×200×50 µm^3^.

### Data collection, structure determination and refinement of GA57BK

Crystals were directly flash frozen in liquid nitrogen. Native diffraction data were collected at the SOLEIL PROXIMA-1 beamline to 2.7 Å resolution. The XDS package [44] was used for all data integration and scaling. The crystals belong to space group C2 with unit cell dimensions a = 163.9 Å, b = 109.6 Å, c = 102.0 Å, β = 120.4° and contain one biological dimer in the asymmetric unit corresponding to a Matthews coefficient value of 3.4 Å^3^/Da [45]. Data collection statistics are shown in Table 2. The structure of GA57BK was determined by molecular replacement with AMoRe [46] implemented in CCP4 [47] using the breakage-reunion domain of the DNA gyrase from *E. coli* [36] (pdb accession code 1AB4) as a search model. Two distinct orientations and positions were found in the asymmetric unit. Structure refinement was carried out with BUSTER-TNT [48] using two-fold non-crystallographic symmetry restraints. Model building was performed manually with the program Coot [49]. Model refinement statistics are summarized in Table 2. The figures were prepared using *PyMol* [50], available at http://pymol.sourceforge.net/. Interface areas were calculated with the PISA server [51].

**Table 2:** Data collection and refinement statistics.

	TopBK *crystal I*	TopBK *crystal II*	GA57BK
Data Collection			
Beamline	ESRF ID23eh1	SOLEILPROXIMA 1	SOLEILPROXIMA 1
Space group	P4_3_2_1_2	P4_3_2_1_2	C2
Unit cell dimensions			
*a*, *b*, *c* (Å)	52.9, 52.9, 190.2	52.8, 52.8, 190.5	163.9, 109.6, 102.0
*α*, *β*, *γ* (°)	90, 90, 90	90, 90, 90	90, 120.4, 90
Wavelength (Å)	0.9762	0.9800	0.9800
Resolution (Å)	14–2.1 (2.3–2.1)^a^	29–1.95 (2.06–1.95)	35–2.7 (2.8–2.7)
R*_sym_* (%)^b^	13.0 (55.0)	7.5 (58.7)	8.6 (72.6)
Redundancy^a^	8.6 (4.0)	7.6 (7.8)	3.5 (3.5)
Completeness (%)^a^	99.1 (86.5)	99.7 (99.2)	98.9 (99.0)
I/sig(I)^a^	13.1 (3.3)	16.9 (3.4)	12.44 (2.24)
Refinement			
Resolution (Å)	14.0–2.1	17.0–1.95	19.9–2.7
No. Reflections	16487	20626	42396
No. Atoms			
Protein	1474	1482	7534
Water	107	147	238
R*_work_*/R*_free_* c	0.214, 0.249	0.210, 0.230	0.192, 0.233
*B*-factors			
Protein	38.3	37.8	52.5
Water	52.6	52.2	57.6
RMSD			
Bond length (Å)	0.004	0.007	0.004
Bond angles (°)	0.835	0.97	0.719
Ramachandran analysis			
Most favored (%)	93.8	92.7	90.2
Additional allowed (%)	5.6	6.7	9.3
Generously allowed (%)	0.0	0.0	0.4
Disallowed (%)	0.6	0.6	0.1

a The values in parentheses are statistics from the highest resolution shell.

b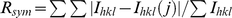 where I*_hkl_*(j) is the jth observed intensity of I_hkl_ and I_hkl_ is the final average value of intensity.

c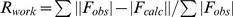 and 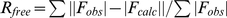 where the sum is restricted to reflections that belong to a test set of 5% randomly selected data.

### Cloning, expression, purification and crystallization of TopBK *crystal I* and *II*

The Toprim domain of DNA gyrase subunit B from *Mycobacterium tuberculosis* (residues 448–675), hereafter named TopBK, was cloned into the expression vector pRSF-2 Ek/LIC (Novagen). The plasmid was transformed into Rosetta 2(DE3) pLysS (Novagen). The transformed cells were grown in LB medium in presence of chloramphenicol and kanamycin. Gene expression was induced by addition of IPTG (Sigma) to a final concentration of 1 mM at 22°C over night. Cells were harvested by centrifugation and stored at −20°C one night. Cells were resuspended in buffer B1 containing 20 mM Tris-HCl pH 8, 500 mM NaCl and 15 mM imidazole. The cells were lysed by sonication. Following the centrifugation, the protein was run over a Ni-NTA column (GE Healthcare) equilibrated with buffer B1 at 4°C. The TopBK protein was eluted using a linear gradient from 15 to 500 mM imidazole. Finally, the protein was loaded on a Superdex-75 10/300 (GE Healthcare) equilibrated with a buffer containing 20 mM Tris-HCl pH 8. The protein was then concentrated to 5 mg/ml in the same buffer.

TopBK *crystal I* was obtained in 10% PEG 4K, 200 mM ammonium sulfate, 15 mM magnesium chloride, 100 mM Tris-HCl pH 8 by vapour diffusion with the hanging drop vapour diffusion method mixing 2 volumes of protein sample with 1 volume of reservoir solution [10% PEG 4K, 200 mM ammonium sulfate, 15 mM magnesium chloride, 100 mM Tris-HCl pH 8]. TopBK *crystal II* was obtained in similar conditions, except that magnesium chloride was substituted by calcium chloride.

### Data collection, structure determination and refinement of TopBK

For TopBK *crystal I*, diffraction data were collected at ESRF on beamline id23eh1 to 2.1 Å resolution. The XDS package was used for data processing and scaling (Table 2). The crystals belong to space group P4_3_2_1_2 with unit cell dimensions a = b = 52.87 Å, c = 190.22 Å. The structure of TopBK was determined by molecular replacement with Molrep [52] implemented in ccp4 using one monomer of the previously published structure (PDB accession code 2ZJT, [24]) as the starting model. The asymmetric unit contains one monomer corresponding to a Matthews coefficient value of 2.4 Å^3^/Da. Structure refinement was carried out with BUSTER-TNT to 2.1 Å resolution. Model building was performed manually with the program coot. Model refinement statistics are summarized in Table 2. For TopBK *crystal II*, diffraction data were collected at SOLEIL PROXIMA 1 to 1.95 Å resolution. TopBK *crystal II* is isomorphous to *crystal I* and structure determination protocol was the same as for *crystal I* (Table 2).

### Analytical ultracentrifugation

Sedimentation velocity experiments were performed in a Beckman XL-I analytical ultracentrifuge using a double sector charcoal-Epon cell at 20°C and 42000 rpm. Absorbance scans were taken at 276 nm every 6 min. The protein concentration was 1 mg/ml for GA57BK corresponding to 17.5 µM in 20 mM Tris pH 8. For TopBK, experiments were performed at three protein concentrations, 0.5, 1 and 4 mg/ml (corresponding to 18, 37 and 148 µM, respectively) in the same buffer. The program Sednterp 1.09 (available at http://www.rasmb.bbri.org) was used to calculate solvent density (0.9988 g/cm^3^), solvent viscosity (0.010069 Poise) and partial specific volume (0.7340 ml/g for GA57BK and 0.7390 for TopBK) using the amino-acid composition. The sedimentation data were analyzed with the program Sedfit [53] using the continuous c(s) and c(M) distributions. Theoretical sedimentation coefficients were calculated from the crystal structure PDB file using Hydropro 7c [54] with a hydrated radius of 3.4 Å for the atomic elements. The same experiments were performed for GA57BK and TopBK in 20 mM Tris pH 8 and 100 mM NaCl. Sedimentation data were analyzed with appropriate values of solvent density and viscosity.

### Activity assays

DNA supercoiling and cleavage assays were carried out as previously described [7], [13], [23], [55]. Briefly, DNA cleavage assays were performed with various ratios of purified *M. tuberculosis* GyrA and GyrB subunits or GA57BK and TopBK domains. The reaction mixture (total volume 20 µl) contained DNA gyrase assay buffer (40 mM Tris-HCl pH 7.5, 25 mM KCl, 6 mM magnesium acetate, 2 mM spermidine, 4 mM DTT, 0.1 mg/ml *E. coli* tRNA, BSA (0.36 mg/ml), 100 mM potassium glutamate), supercoiled pBR322 DNA (0.4 µg) as the substrate and moxifloxacin (50 µg/ml). Proteins were added and reaction mixtures were incubated at 25°C for 1 h. Three µl of 2% SDS and 3 µl of a 1 mg/ml solution of proteinase K were added, and incubation was continued for 30 min at 37°C. Reactions were terminated by the addition of 50% glycerol containing 0.25% bromophenol blue, and the total reaction mixture was subjected to electrophoresis in 1% agarose gel in TBE 0.5× buffer (Tris-Borate-EDTA, pH 8.3). After running for 3.5 hrs at 50 V, the gel was stained with ethidium bromide (0.7 µg/ml), photographed and quantified with an Alpha Innotech digital camera and associated software. All enzyme assays were done at least twice, with reproducible results.

### Molecular modeling

The catalytic core model (GA57BK_2_+TopBK_2_+DNA) was generated by superposition onto the crystal structure of the *Streptococcus pneumoniae* topoisomerase IV catalytic core [26] (pdb accession code 3FOF). Chains A and B from 3IFZ (GA57BK) were superposed to the corresponding chains from 3FOF, respectively, using SSM implemented in coot. The two disordered regions of the TopBK structure were modeled using the Toprim domain of 3K9F as a template. The amino acid torsion angles in these regions were validated using the Ramachandran plot. The two monomers of TOPBK were superposed using the same method to the chains C and D of the *S. pneumoniae* topoisomerase IV catalytic core structure. The DNA coordinates (chain E, F, G, H) without moxifloxacin were inserted in the complex and defined as fixed atoms. The complex was then energy minimized. Energy minimization was performed with the NAMD2 program [56] using CHARMM27 force field. The system was minimized by 300 000 steps of conjugate gradient minimization. Non bonded interaction parameters were set such that electrostatic interaction is shifted to zero at 12 Å and the van der Waals interaction is switched off from 10 Å to 12 Å.

For the docking, the two fluoroquinolone moieties were extracted from 3FOF coordinates. They were positioned in the minimized catalytic core with respect to their respective positions in the 3FOF structure. The system was further minimized using the Minimization module of Discovery Studio© (Accelrys), the CHARMM forcefield and a cascade of Steepest Descent, Gradient Conjugate and Adopted Basis Newton Raphson minimizations, during which the backbone of the protein complex plus the DNA atoms were constrained while the side chains and ligand moieties were allowed to relax (6,000 iterations with final RMS gradient 0.01). We computed energetic criteria as the potential energy of the complex. The minimised model deviates from the crystal structure of the *Streptococcus pneumoniae* topoisomerase IV catalytic core with an rmsd of 2.4 Å over 1033 Cα atoms. Finally, we computed the interaction energy (which corresponds to the sum of VDW and electrostatics non-bonded interactions) between each moxifloxacin and its devoted quinolone-binding pocket with the Calculate Interaction Energy module of Discovery Studio© (Accelrys).

### Accession numbers

Co-ordinates and structure factors of TopBK *crystal I* have been deposited in the protein data bank with the code 3IG0, TopBK *crystal II* with the code 3M4I and GA57BK with the code 3IFZ.

## Supporting Information

Table S1 Values of the interfaces calculated by PISA for the five structures of the breakage-reunion domain dimer in closed conformation. The PDB codes for the five structures are given: 3IFZ (this work) and 3ILW (25) correspond to *M. tuberculosis* DNA gyrase, 1AB4 (36) to *E. coli* DNA gyrase, 2INR (34) to *S. aureus* topoisomerase IV, 2NOV (33) to *S. pneumoniae* topoisomerase IV. Nat, Nres correspond to the number of atoms and residues, respectively, in interaction between the two monomers.
(1.36 MB DOC)

Figure S1 Structure-based sequence alignment of the Toprim domain from type II topoisomerases. The sequence names are as follows: MtGyr (PDB code 3IFZ) (this work), *M. tuberculosis* DNA gyrase; SpTopIV (PDB code 3FOF) (26), *S. pneumoniae* topoisomerase IV and ScTopII (PDB code 2RGR) (29), *S. cerevisiae* topoisomerase II. alpha-helices (cylinders) and beta-strands (arrows) of *M. tuberculosis* GA57BK are shown with the sequences and color-coded according to Figure 1 (Toprim region in yellow, the hinge in blue and the Tail region in purple). Residues emphasized by black shading are 100% conserved. The magnesium binding site residues are underlined by red stars (E and DxD). The disordered regions are emphasized in pale grey and indicated as alpha1 and DBL for DNA Binding Loop. The QRDR-B is delimited by a blue frame.
(0.04 MB DOC)

Figure S2 The three different conformations of the breakage-reunion domain. A. The breakage-reunion domain of *M. tuberculosis* (PDB id 3IFZ) (this work), representing the closed conformation with the DNA-gate and the C-gate closed. This closed conformation is also observed in the *E. coli* DNA gyrase (36), *S. pneumoniae* and *S. aureus* topoisomerase IV breakage-reunion domain structures (33,34). B. The breakage-reunion domain of *S. cerevisiae* in complex with DNA (PDB id 2RGR) (29), representing an open conformation with the DNA-gate open and the C-gate closed. C. The breakage-reunion domain of *S. cerevisiae* (PDB id 1BGW) (31), representing an open conformation with the DNA-gate closed and the C-gate open.
(0.90 MB DOC)

Figure S3 Structure-based sequence alignment of the breakage-reunion domain from type II topoisomerases. The sequence names are as follows: MtGyr (PDB code 3IFZ) (this work), *M. tuberculosis* DNA gyrase; EcGyr (PDB code 1AB4) (36), *E. coli* DNA gyrase; SaTopIV (PDB code 2INR) (34), *S. aureus* topoisomerase IV; SpTopIV (PDB code 2NOV) (33), *S. pneumoniae* topoisomerase IV; EcTopIV (PDB code 1ZVU), *E. coli* topoisomerase IV and ScTopII (PDB code 2RGR) (29), *S. cerevisiae* topoisomerase II. alpha-helices (cylinders) and beta-strands (arrows) of *M. tuberculosis* GA57BK are shown with the sequences and color-coded according to Figure 1 (N-terminal helix in red, DNA-gate in blue, Tower in green, helix bundle in orange and C-gate in purple). Residues emphasized by black shading are 100% conserved. The catalytic residues are underlined by red stars (R128 and Y129) and GA57BK specific motifs by black stars (the DPP and DEEX motifs). The QRDR-A is delimited by a blue frame.
(0.06 MB DOC)

Figure S4 Superimposition of the different monomer structures of the breakage-reunion domain. *M. tuberculosis* DNA gyrase GA57BK (3IFZ) (this work) in light green, *M. tuberculosis* DNA gyrase MtGyrA59 (3ILW, 25) in pale green, *E. coli* DNA gyrase (1AB4) (36) in dark green, *S. pneumoniae* topoisomerase IV (2NOV) (33) in red, *S. aureus* topoisomerase IV (2INR) (34) in pale red, *S. pneumoniae* complexed with DNA (3FOF) (26) in dark red and *E. coli* topoisomerase IV (1ZVU) in firebrick. The rmsd (in Ang.) after superimposition and the number of common Cα (in parenthesis) are indicated in the table. The color code is conserved.
(0.43 MB DOC)
