# Supplementary material for: Structural Insights into the Quinolone Resistance Mechanism of Mycobacterium tuberculosis DNA Gyrase
Source: PLoS One. 2010 Aug 18;5(8):e12245. doi: 10.1371/journal.pone.0012245 (PMC2923608; doi:10.1371/journal.pone.0012245)
Supplement: Table S1 — Values of the interfaces calculated by PISA for the five structures of the breakage-reunion domain dimer in closed conformation. The PDB codes for the five structures are given: 3IFZ (this work) and 3ILW (25) correspond to M. tuberculosis DNA gyrase, 1AB4 (36) to E. coli DNA gyrase, 2INR (34) to S. aureus topoisomerase IV, 2NOV (33) to S. pneumoniae topoisomerase IV. Nat, Nres correspond to the number of atoms and residues, respectively, in interaction between the two monomers. (1.36 MB DOC) [file pone.0012245.s001.doc]

|  |  | Interface on the DNA gate | | | Interface on the C-gate | | | Total interface | | |
| --- | --- | --- | --- | --- | --- | --- | --- | --- | --- | --- |
| **PDB** | **Protein** | **N_at_** | **N_res_** | **Interface Å^2^** | **N_at_** | **N_res_** | **Interface Å^2^** | **N_at_** | **N_res_** | **Interface Å^2^** |
| **3IFZ** | ***Mt*Gyr** | 82 | 23 | 828.6 | 112 | 26 | 1120.3 | 195 | 49 | 1952.3 |
| **3ILW** | ***Mt*Gyr** | 81 | 22 | 822.9 | 116 | 27 | 1157.6 | 198 | 50 | 1980.5 |
| **1AB4** | ***Ec*Gyr** | 73 | 19 | 684.4 | 104 | 27 | 1100.6 | 177 | 46 | 1784.7 |
| **2INR** | ***Sa*TopIV** | 58 | 17 | 584.5 | 101 | 25 | 1038.5 | 159 | 42 | 1622.9 |
| **2NOV** | ***Sp*TopIV** | 39 | 15 | 384.3 | 96 | 23 | 1029.4 | 153 | 39 | 1415.8 |

**Table S1.** Values of the interfaces calculated by PISA for the five structures of the breakage-reunion domain dimer in closed conformation. The PDB codes for the five structures are given: 3IFZ (this work) and 3ILW (25) correspond to *M. tuberculosis* DNA gyrase, 1AB4 (36) to *E. coli* DNA gyrase, 2INR (34) to *S. aureus* topoisomerase IV, 2NOV (33) to *S. pneumoniae* topoisomerase IV. N_at_, N_res_ correspond to the number of atoms and residues, respectively, in interaction between the two monomers.

α2

β1

β2

α1

DBL

***Mt*Gyr** PRKS ELYVVEGDSAGGSAKSGR---DSMFQAILPLRGKIINVEKARIDRVLKNTE VQAIITALGTGIHDEFD

***Sp*TopIV** PAKN ELYLVEGDSAGGSAKQGR---DRKFQAILPLRGKVINTAKAKMADILKNEE INTMIYTIGAGVGADFS

***Sc*TopII** GYKC TLVLTEGDSALSLAVAGLAVVGRDYYGCYPLRGKMLNVREASADQILKNAE IQAIKKIMGLQHRKKYE

*****

β3

β4

β5

β6

α3

α4

α4

***Mt*Gyr** -IGKLRYHKIVLMADADVDGQHISTLLLTLLFRFMR-PLIENGHVFLAQPPLYKLKWQ---RSDPEFAYSDRER

***Sp*TopIV** -IEDANYDKIIIMTDADTDGAHIQTLLLTFFYRYMR-PLVEAGHVYIALPPLYKMSKGKGKKEEVAYAWTDGEL

***Sc*TopII** DTKSLRYGHLMIMTDQDHDGSHIKGLIINFLESSFLGLLDIQGFLLEFITPIIKVSITKPTKNTIAFYNMPDYE

* *

α4

α5

α6

β7

β8

***Mt*Gyr** DGLLEAGLKAGKKINKEDGIQRYKGLGEMDAKELWETTMDPSVRVLRQVTLDDAAAADELFSILMG-EDVDAR

***Sp*TopIV** EELRKQF---GKGA----TLQRYKGLGEMNADQLWETTMNPETRTLIRVTIEDLARAERRVNVLMG-DKVEPR

***Sc*TopII** KWREEESHK-KFTW----KQKYYKGLGTSLAQEVREYFSNLDRHLKIFHSLQGNDKDYIDLAFSKKKADDRKE

***Mt*Gyr** RSFITRNAKDVRFLDVKLAAAL

***Sp*TopIV** RKWIEDNVK----FTLEEATV-

***Sc*TopII** --WLRQYEPGTVLDPTLKEIP-

**Figure S1.** Structure-based sequence alignment of the Toprim domain from type II topoisomerases. The sequence names are as follows: ***Mt*Gyr** (PDB code 3IFZ) (this work), *M. tuberculosis* DNA gyrase; ***Sp*TopIV** (PDB code 3FOF) (26)**,** *S. pneumoniae* topoisomerase IV and ***Sc*TopII** (PDB code 2RGR) (29), *S. cerevisiae* topoisomerase II. α-helices (cylinders) and β-strands (arrows) of *M. tuberculosis* GA57BK are shown with the sequences and color-coded according to Figure 1 (Toprim region in yellow, the hinge in blue and the Tail region in purple). Residues emphasized by black shading are 100 % conserved. The magnesium binding site residues are underlined by red stars (E and DxD). The disordered regions are emphasized in pale grey and indicated as α1 and DBL for DNA Binding Loop. The QRDR-B is delimited by a blue frame.


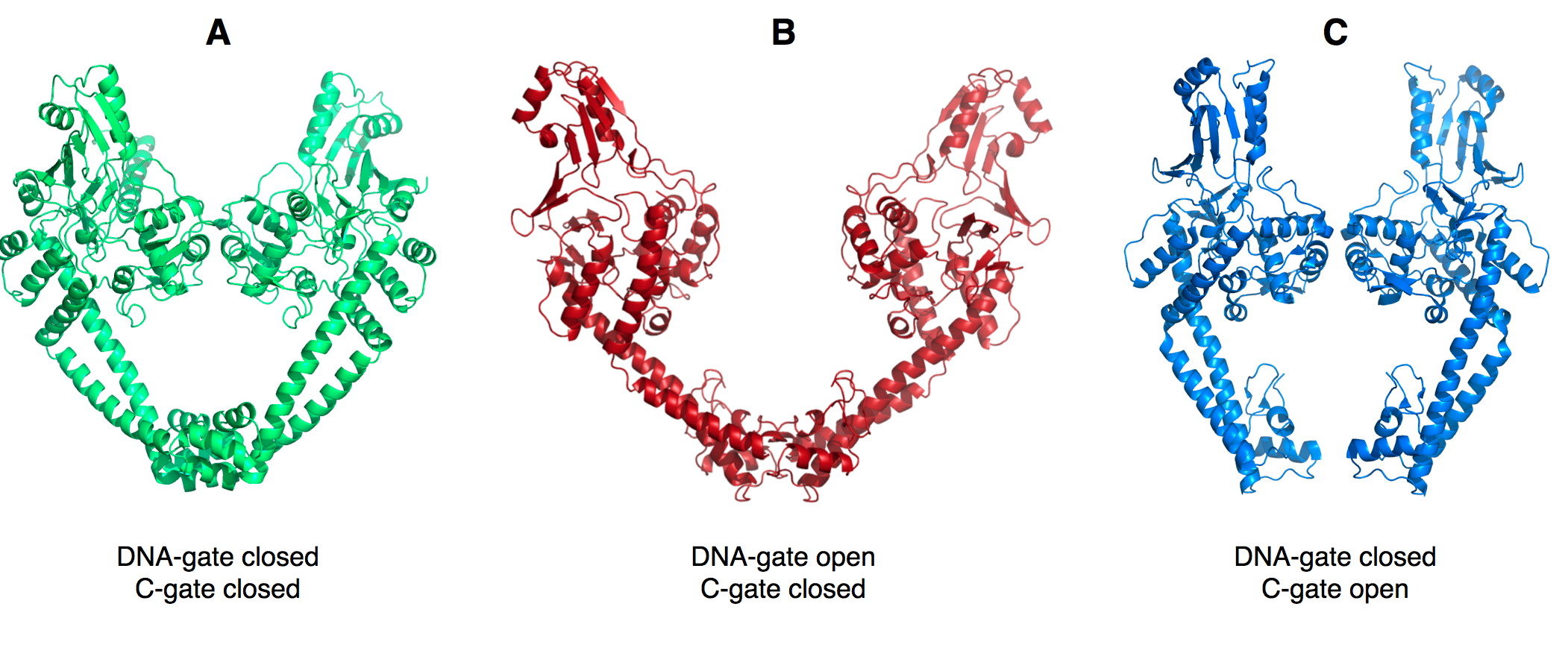


**Figure S2.** The three different conformations of the breakage-reunion domain. **A.** The breakage-reunion domain of *M. tuberculosis* (PDB id 3IFZ) (this work), representing the closed conformation with the DNA-gate and the C-gate closed. This closed conformation is also observed in the *E. coli* DNA gyrase (36), *S. pneumoniae* and *S. aureus* topoisomerase IV breakage-reunion domain structures (33,34). **B.** The breakage-reunion domain of *S. cerevisiae* in complex with DNA (PDB id 2RGR) (29), representing an open conformation with the DNA-gate open and the C-gate closed. **C.** The breakage-reunion domain of *S. cerevisiae* (PDB id 1BGW) (31), representing an open conformation with the DNA-gate closed and the C-gate open.

α1

α2

***Mt*Gyr** ..MTDTTLPPDDSLDRIEPVDIEQEMQRSYIDYAMSVIVGRALPEVRDGLKPVHRRVLYAMFDSGFRPDRSHA

***Ec*Gyr**  .........MSDLAREITPVNIEEELKSSYLDYAMSVIVGRALPDVRDGLKPVHRRVLYAMNVLGNDWNKAYK

***Sa*TopIV** ............MSEIIQDLSLEDVLGDRFGRYSKYIIQERALPDVRDGLKPVQRRILYAMYSSGNTHDKNFR

***Sp*TopIV** .............MSNIQNMSLEDIMGERFGRYSKYIIQDRALPDIRDGLKPVQRRILYSMNKDSNTFDKSYR

***Ec*TopIV** ............MSDMAERLALHEFTENAYLNYSMYVIMDRALPFIGDGLKPVQRRIVYAMSELGLNASAKFK

***Sc*TopII** .......................SDFINKELILFSLADNIRSIPNVLDGFKPGQRKVLYGCFKKNLK...SEL

α3

α4

α7

β2

β3

***Mt*Gyr** KQSARSVAETMGNY.HPHGDASIYDSLVRMAQPW..SLRYPLVDGQ GNFGSPGN..DPPAAMRYTEARLTPLAM

***Ec*Gyr**  K.SARVVGDVIGKY.HPHGDSAVYDTIVRMAQPF..SLRYMLVDGQ GNFGSIDG..DSAAAMRYTEIRLAKIAH

***Sa*TopIV** K.SAKTVGDVIGQY.HPHGDSSVYEAMVRLSQDW..KLRHVLIEMH GNNGSIDN..DPPAAMRYTEAKLSLLAE

***Sp*TopIV** K.SAKSVGNIMGNF.HPHGDSSIYDAMVRMSQNW..KNREILVEMH GNNGSMDG..DPPAAMRYTEARLSEIAG

***Ec*TopIV** K.SARTVGDVLGKY.HPHGDSACYEAMVLMAQPF..SYRYPLVDGQ GNWGAPDDP.KSFAAMRYTESRLSKYSE

***Sc*TopII** K.VAQLAPYVSECTAYHHGEQSLAQTIIGLAQNFVGSNNIYLLLPN GAFGTRATGGKDAAAARYIYTELNKLTR

******* ******

α10

α9

α7

β4

β5

β6

β7

***Mt*Gyr** EMLR.E.IDEETVDFIPNYDGRVQEPTVLPSRFPNLLANGSGGIAVGMATNIPPHNLRELADAVFWALENHDA

***Ec*Gyr**  ELMA.D.LEKETVDFVDNYDGTEKIPDVMPTKIPNLLVNGSSGIAVGMATNIPPHNLTEVINGCLAYIDDEDI

***Sa*TopIV** ELLR.D.INKETVSFIPNYDDTTLEPMVLPSRFPNLLVNGSTGISAGYATDIPPHNLAEVIQATLKYIDNPDI

***Sp*TopIV** YLLQ.D.IEKKTVPFAWNFDDTEKEPTVLPAAFPNLLVNGSTGISAGYATDIPPHNLAEVIDAAVYMIDHPTA

***Ec*TopIV** LLLS.E.LGQGTADWVPNFDGTLQEPKMLPARLPNILLNGTTGIAVGMATDIPPHNLREVAQAAIALIDQPKT

***Sc*TopII** KIFHPADD..PLYKYIQE.DEKTVEPEWYLPILPMILVNGAEGIGTGWSTYIPPFNPLEIIKNIRHLMNDEEL

α10’

α10’’

β10

β9

β11

β7’

***Mt*Gyr** DEEETLAAVMGRVKGPDFPTA.GLIVG...SQGTADAYKTGRGSIRMRGVVEVEE.DSRGRTSLVITELPYQV

***Ec*Gyr**  ....SIEGLMEHIPGPDFPTA.AIING...RRGIEEAYRTGRGKVYIRARAEVEVDAKTGRETIIVHEIPYQV

***Sa*TopIV** ....TVNQLMKYIKGPDFPTG.GIIQG...IDGIKKAYESGKGRIIVRSKVEEET.LRNGRKQLIITEIPYEV

***Sp*TopIV** ....KIDKLMEFLPGPDFPTG.AIIQG...RDEIKKAYETGKGRVVVRSKTEIEK.LKGGKEQIVITEIPYEI

***Ec*TopIV** ....TLDQLLDIVQGPDYPTE.AEIIT...SRAEIRKIYENGRGSVRMRAVWKKED.....GAVVISALPHQV

***Sc*TopII** ........EQ...MHPWFRGWTGTIEEIEP.............LRYRMYGRIEQIGD....NVLEITELPART

********

α11

α12

β12

β13

β14

β15

***Mt*Gyr** NHDNFITSIAEQVRDGKLAGI..SNIEDQSSDRVGLRIVIEIKRDAVAKVVI.NNLYKHTQLQTSFGA.NMLA

***Ec*Gyr**  NKARLIEKIAELVKEKRVEGI..SALRDES.DKDGMRIVIEVKRDAVGEVVL.NNLYSQTQLQVSFGI.NMVA

***Sa*TopIV** NKSSLVKRIDELRADKKVDGI..VEVRDET.DRTGLRIAIELKKDVNSESIK.NYLYKNSDLQISYNF.NMVA

***Sp*TopIV** NKANLVKKIDDVRVNNKVAGI..AEVRDES.DRDGLRIAIELKKDANTELVL.NYLFKYTDLQINYNF.NMVA

***Ec*TopIV** SGARVLEQIAAQMRNKKLPMV..DDLRDESDHENPTRLVIVPRSNRVDMDQVMNHLFATTDLEKSYRINLNMI

***Sc*TopII** WTSTIKEYLLLGLSGNDKIKPWIKDMEEQH.D.DNIKFIITLSPEEMAKTRK.IGFYERFKLISPISLMNMVA

α14

α14’

α15

β15

β16

***Mt*Gyr** IV.DGVPRTL.RLDQLIRYYVDHQLDVIVRRTTYRLRKANERAHILRGLVKALDAL..DEVIALI.RASETVD

***Ec*Gyr**  LH.HGQPKIM.NLKDIIAAFVRHRREVVTRRTIFELRKARDRAHILEALAVALANI..DPIIELI.RHAPTPA

***Sa*TopIV** IS.DGRPKLM.GIRQIIDSYLNHQIEVVANRTKFELDNAEKRMHIVEGLIKALSIL..DKVIELI.RSSKNKR

***Sp*TopIV** ID.NFTPRQV.GIVPILSSYIAHRREVILARSRFDKEKAEKRLHIVEGLIRVISIL..DEVIALI.RASENKA

***Ec*TopIV** GLDGRPAVKN.LLE.ILSEWLVFRRDTVRRRLNYRLEKVLKRLHILEGLLVAFLNI..DEVIEII.R...NED

***Sc*TopII** FDPHGKIKKYNSVNEILSEFYYVRLEYYQKRKDHMSERLQWEVEKYSFQVKFIKMIIEKELT..VTNKP..RN

α15

α15

***Mt*Gyr** IARAGLIEL..................................LDID..........................

***Ec*Gyr**  EAKTALVANPWQLGNVAAMLERAGDDAARPEWLEPEFGVRDGLYYLT..........................

***Sa*TopIV** DAKENLIEV..................................YEFT..........................

***Sp*TopIV** DAKENLKVS..................................YDFT..........................

***Ec*TopIV** EPKPALMSR..................................FGLT..........................

***Sc*TopII** AIIQELENLG.................................FPRFNKEGKPYYGSPNDEIAEQINDVKGAT

α19

α16

α18

***Mt*Gyr** .......................EIQAQAILDMQLRRLAALERQRIIDDLAKIEAEIADLEDILAKPERQRGI

***Ec*Gyr**  .......................EQQAQAILDLRLQKLTGLEHEKLLDEYKELLDQIAELLRILGSADRLMEV

***Sa*TopIV** .......................EEQAEAIVMLQLYRLTNTDIVALEGEHKELEALIKQLRHILDNHDALLNV

***Sp*TopIV** .......................EEQAEAIVTLQLYRLTNTDVVVLQEEEAELREKIAMLAAIIGDERTMYNL

***Ec*TopIV** .......................ETQAEAILELKLRHLAKLEEMKIRGEQSELEKERDQLQGILASERKMNNL

***Sc*TopII** SDEEDEESSHEDTENVINGPEELYGTYEYLLGMRIWSLTKERYQKLLKQKQEKETELENLLKL..SAKDIWNT

α19

β20

***Mt*Gyr** VRDELAEIVDRHGDDRRTRIIAA.....

***Ec*Gyr**  IREELELVREQFGDKRRTEIT.......

***Sa*TopIV** IKEELNEIKKKFKSERLSLIEAEIEE..

***Sp*TopIV** MKKELREVKKKFATPRL...........

***Ec*TopIV** LKKELQADAQAYGDDRRSPLQEREEAKA

***Sc*TopII** DLKAFEVGYQEFLQRDAEAR........

**Figure S3.** Structure-based sequence alignment of the breakage-reunion domain from type II topoisomerases. The sequence names are as follows: ***Mt*Gyr** (PDB code 3IFZ) (this work), *M. tuberculosis* DNA gyrase; ***Ec*Gyr** (PDB code 1AB4) (36)**,** *E. coli* DNA gyrase*;* ***Sa*TopIV** (PDB code 2INR) (34)**,** *S. aureus* topoisomerase IV; ***Sp*TopIV** (PDB code 2NOV) (33)**,** *S. pneumoniae* topoisomerase IV; ***Ec*TopIV** (PDB code 1ZVU) (see below)**,** *E. coli* topoisomerase IV and ***Sc*TopII** (PDB code 2RGR) (29), *S. cerevisiae* topoisomerase II. α-helices (cylinders) and β-strands (arrows) of *M. tuberculosis* GA57BK are shown with the sequences and color-coded according to Fig 1 (N-terminal helix in red, DNA-gate in blue, Tower in green, helix bundle in orange and C-gate in purple). Residues emphasized by black shading are 100 % conserved. The catalytic residues are underlined by red stars (R128 and Y129) and GA57BK specific motifs by black stars (the DPP and DEEX motifs). The QRDR-A is delimited by a blue frame.

**Reference of PDB code 1ZVU**

Corbett, K.D.,   Schoeffler, A.J.,   Thomsen, N.D.,   Berger, J.M. (2005). The structural basis for substrate specificity in DNA topoisomerase IV. J.Mol.Biol. **351:** 545-561.


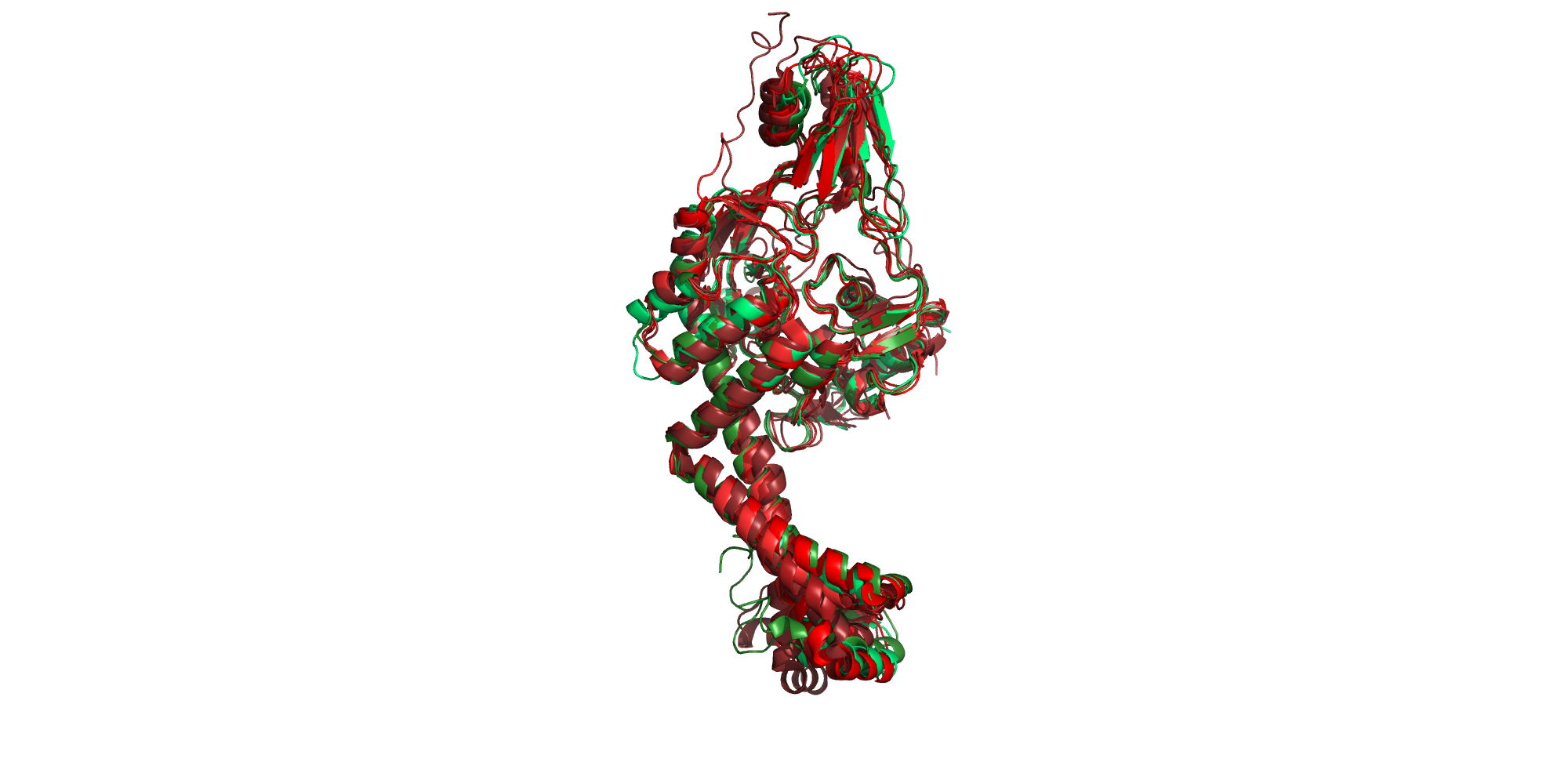


|  | **3ILW**  ***Mt*Gyr** | **1AB4**  ***Ec*Gyr** | **2NOV**  ***Sp*TopIV** | **2INR**  ***Sa*TopIV** | **3FOF**  ***Sp*TopIV** | **1ZVU**  ***Sc*TopIV** |
| --- | --- | --- | --- | --- | --- | --- |
| **3IFZ *Mt*Gyr** | 0.543 (354) | 0.847 (368) | 1.372 (399) | 0.869 (351) | 1.426 (362) | 1.576 (321) |
| **3ILW**  ***Mt*Gyr** |  | 0.991 (384) | 1.934 (396) | 0.969 (370) | 1.317 (353) | 1.796 (336) |
| **1AB4 *Ec*Gyr** |  |  | 1.813 (395) | 1.365 (387) | 1.577 (365) | 1.546 (317) |
| **2NOV *Sp*TopIV** |  |  |  | 0.904 (347) | 1.080 (339) | 1.884 (318) |
| **2INR *Sa*TopIV** |  |  |  |  | 0.867 (359) | 1.813 (326) |
| **3FOF *Sp*TopIV** |  |  |  |  |  | 2.597 (310) |

**Figure S4**. Superimposition of the different monomer structures of the breakage-reunion domain. *M. tuberculosis* DNA gyrase GA57BK (3IFZ) (his work) in light green, *M. tuberculosis* DNA gyrase *Mt*GyrA59 (3ILW, 25) in pale green, *E. coli* DNA gyrase (1AB4) (36) in dark green*, S. pneumoniae* topoisomerase IV (2NOV) (33) in red*, S. aureus* topoisomerase IV (2INR) (34) in pale red, *S. pneumoniae* complexed with DNA (3FOF) (26) in dark red and *E. coli* topoisomerase IV (1ZVU) (see below) in firebrick. The rmsd (in Å) after superimposition and the number of common C_α_ (in parenthesis) are indicated in the table. The color code is conserved.

**Additional reference**

Corbett KD, Schoeffler AJ, Thomsen ND, Berger JM (2005). The structural basis for substrate specificity in DNA topoisomerase IV. J Mol Biol. 351: 545-561.
