# Supplementary material for: Structural Insights into the Quinolone Resistance Mechanism of Mycobacterium tuberculosis DNA Gyrase
Source: PLoS One. 2010 Aug 18;5(8):e12245. doi: 10.1371/journal.pone.0012245 (PMC2923608; doi:10.1371/journal.pone.0012245)
Supplement: Figure S1 — Structure-based sequence alignment of the Toprim domain from type II topoisomerases. The sequence names are as follows: MtGyr (PDB code 3IFZ) (this work), M. tuberculosis DNA gyrase; SpTopIV (PDB code 3FOF) (26), S. pneumoniae topoisomerase IV and ScTopII (PDB code 2RGR) (29), S. cerevisiae topoisomerase II. alpha-helices (cylinders) and beta-strands (arrows) of M. tuberculosis GA57BK are shown with the sequences and color-coded according to Figure 1 (Toprim region in yellow, the hinge in blue and the Tail region in purple). Residues emphasized by black shading are 100% conserved. The magnesium binding site residues are underlined by red stars (E and DxD). The disordered regions are emphasized in pale grey and indicated as alpha1 and DBL for DNA Binding Loop. The QRDR-B is delimited by a blue frame. (0.04 MB DOC) [file pone.0012245.s002.doc]

α2

β1

β2

α1

DBL

***Mt*Gyr** PRKS ELYVVEGDSAGGSAKSGR---DSMFQAILPLRGKIINVEKARIDRVLKNTE VQAIITALGTGIHDEFD

***Sp*TopIV** PAKN ELYLVEGDSAGGSAKQGR---DRKFQAILPLRGKVINTAKAKMADILKNEE INTMIYTIGAGVGADFS

***Sc*TopII** GYKC TLVLTEGDSALSLAVAGLAVVGRDYYGCYPLRGKMLNVREASADQILKNAE IQAIKKIMGLQHRKKYE

*****

β3

β4

β5

β6

α3

α4

α4

***Mt*Gyr** -IGKLRYHKIVLMADADVDGQHISTLLLTLLFRFMR-PLIENGHVFLAQPPLYKLKWQ---RSDPEFAYSDRER

***Sp*TopIV** -IEDANYDKIIIMTDADTDGAHIQTLLLTFFYRYMR-PLVEAGHVYIALPPLYKMSKGKGKKEEVAYAWTDGEL

***Sc*TopII** DTKSLRYGHLMIMTDQDHDGSHIKGLIINFLESSFLGLLDIQGFLLEFITPIIKVSITKPTKNTIAFYNMPDYE

* *

α4

α5

α6

β7

β8

***Mt*Gyr** DGLLEAGLKAGKKINKEDGIQRYKGLGEMDAKELWETTMDPSVRVLRQVTLDDAAAADELFSILMG-EDVDAR

***Sp*TopIV** EELRKQF---GKGA----TLQRYKGLGEMNADQLWETTMNPETRTLIRVTIEDLARAERRVNVLMG-DKVEPR

***Sc*TopII** KWREEESHK-KFTW----KQKYYKGLGTSLAQEVREYFSNLDRHLKIFHSLQGNDKDYIDLAFSKKKADDRKE

***Mt*Gyr** RSFITRNAKDVRFLDVKLAAAL

***Sp*TopIV** RKWIEDNVK----FTLEEATV-

***Sc*TopII** --WLRQYEPGTVLDPTLKEIP-

**Figure S1.** Structure-based sequence alignment of the Toprim domain from type II topoisomerases. The sequence names are as follows: ***Mt*Gyr** (PDB code 3IFZ) (this work), *M. tuberculosis* DNA gyrase; ***Sp*TopIV** (PDB code 3FOF) (26)**,** *S. pneumoniae* topoisomerase IV and ***Sc*TopII** (PDB code 2RGR) (29), *S. cerevisiae* topoisomerase II. α-helices (cylinders) and β-strands (arrows) of *M. tuberculosis* GA57BK are shown with the sequences and color-coded according to Figure 1 (Toprim region in yellow, the hinge in blue and the Tail region in purple). Residues emphasized by black shading are 100 % conserved. The magnesium binding site residues are underlined by red stars (E and DxD). The disordered regions are emphasized in pale grey and indicated as α1 and DBL for DNA Binding Loop. The QRDR-B is delimited by a blue frame.
