# Supplementary material for: Structural Insights into the Quinolone Resistance Mechanism of Mycobacterium tuberculosis DNA Gyrase
Source: PLoS One. 2010 Aug 18;5(8):e12245. doi: 10.1371/journal.pone.0012245 (PMC2923608; doi:10.1371/journal.pone.0012245)
Supplement: Figure S2 — The three different conformations of the breakage-reunion domain. A. The breakage-reunion domain of M. tuberculosis (PDB id 3IFZ) (this work), representing the closed conformation with the DNA-gate and the C-gate closed. This closed conformation is also observed in the E. coli DNA gyrase (36), S. pneumoniae and S. aureus topoisomerase IV breakage-reunion domain structures (33,34). B. The breakage-reunion domain of S. cerevisiae in complex with DNA (PDB id 2RGR) (29), representing an open conformation with the DNA-gate open and the C-gate closed. C. The breakage-reunion domain of S. cerevisiae (PDB id 1BGW) (31), representing an open conformation with the DNA-gate closed and the C-gate open. (0.90 MB DOC) [file pone.0012245.s003.doc]

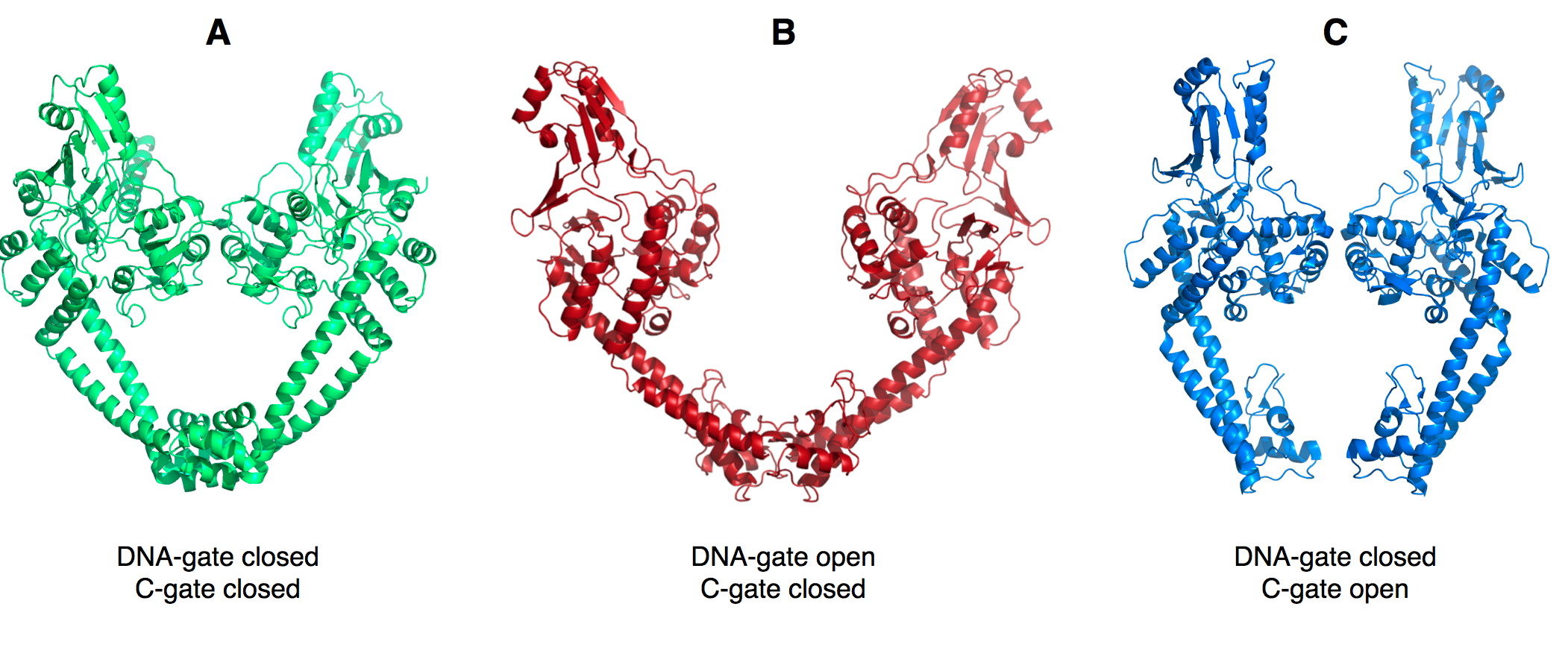


**Figure S2.** The three different conformations of the breakage-reunion domain. **A.** The breakage-reunion domain of *M. tuberculosis* (PDB id 3IFZ) (this work), representing the closed conformation with the DNA-gate and the C-gate closed. This closed conformation is also observed in the *E. coli* DNA gyrase (36), *S. pneumoniae* and *S. aureus* topoisomerase IV breakage-reunion domain structures (33,34). **B.** The breakage-reunion domain of *S. cerevisiae* in complex with DNA (PDB id 2RGR) (29), representing an open conformation with the DNA-gate open and the C-gate closed. **C.** The breakage-reunion domain of *S. cerevisiae* (PDB id 1BGW) (31), representing an open conformation with the DNA-gate closed and the C-gate open.
