# Supplementary material for: Structural Insights into the Quinolone Resistance Mechanism of Mycobacterium tuberculosis DNA Gyrase
Source: PLoS One. 2010 Aug 18;5(8):e12245. doi: 10.1371/journal.pone.0012245 (PMC2923608; doi:10.1371/journal.pone.0012245)
Supplement: Figure S3 — Structure-based sequence alignment of the breakage-reunion domain from type II topoisomerases. The sequence names are as follows: MtGyr (PDB code 3IFZ) (this work), M. tuberculosis DNA gyrase; EcGyr (PDB code 1AB4) (36), E. coli DNA gyrase; SaTopIV (PDB code 2INR) (34), S. aureus topoisomerase IV; SpTopIV (PDB code 2NOV) (33), S. pneumoniae topoisomerase IV; EcTopIV (PDB code 1ZVU), E. coli topoisomerase IV and ScTopII (PDB code 2RGR) (29), S. cerevisiae topoisomerase II. alpha-helices (cylinders) and beta-strands (arrows) of M. tuberculosis GA57BK are shown with the sequences and color-coded according to Figure 1 (N-terminal helix in red, DNA-gate in blue, Tower in green, helix bundle in orange and C-gate in purple). Residues emphasized by black shading are 100% conserved. The catalytic residues are underlined by red stars (R128 and Y129) and GA57BK specific motifs by black stars (the DPP and DEEX motifs). The QRDR-A is delimited by a blue frame. (0.06 MB DOC) [file pone.0012245.s004.doc]

α1

α2

***Mt*Gyr** ..MTDTTLPPDDSLDRIEPVDIEQEMQRSYIDYAMSVIVGRALPEVRDGLKPVHRRVLYAMFDSGFRPDRSHA

***Ec*Gyr**  .........MSDLAREITPVNIEEELKSSYLDYAMSVIVGRALPDVRDGLKPVHRRVLYAMNVLGNDWNKAYK

***Sa*TopIV** ............MSEIIQDLSLEDVLGDRFGRYSKYIIQERALPDVRDGLKPVQRRILYAMYSSGNTHDKNFR

***Sp*TopIV** .............MSNIQNMSLEDIMGERFGRYSKYIIQDRALPDIRDGLKPVQRRILYSMNKDSNTFDKSYR

***Ec*TopIV** ............MSDMAERLALHEFTENAYLNYSMYVIMDRALPFIGDGLKPVQRRIVYAMSELGLNASAKFK

***Sc*TopII** .......................SDFINKELILFSLADNIRSIPNVLDGFKPGQRKVLYGCFKKNLK...SEL

α3

α4

α7

β2

β3

***Mt*Gyr** KQSARSVAETMGNY.HPHGDASIYDSLVRMAQPW..SLRYPLVDGQ GNFGSPGN..DPPAAMRYTEARLTPLAM

***Ec*Gyr**  K.SARVVGDVIGKY.HPHGDSAVYDTIVRMAQPF..SLRYMLVDGQ GNFGSIDG..DSAAAMRYTEIRLAKIAH

***Sa*TopIV** K.SAKTVGDVIGQY.HPHGDSSVYEAMVRLSQDW..KLRHVLIEMH GNNGSIDN..DPPAAMRYTEAKLSLLAE

***Sp*TopIV** K.SAKSVGNIMGNF.HPHGDSSIYDAMVRMSQNW..KNREILVEMH GNNGSMDG..DPPAAMRYTEARLSEIAG

***Ec*TopIV** K.SARTVGDVLGKY.HPHGDSACYEAMVLMAQPF..SYRYPLVDGQ GNWGAPDDP.KSFAAMRYTESRLSKYSE

***Sc*TopII** K.VAQLAPYVSECTAYHHGEQSLAQTIIGLAQNFVGSNNIYLLLPN GAFGTRATGGKDAAAARYIYTELNKLTR

******* ******

α10

α9

α7

β4

β5

β6

β7

***Mt*Gyr** EMLR.E.IDEETVDFIPNYDGRVQEPTVLPSRFPNLLANGSGGIAVGMATNIPPHNLRELADAVFWALENHDA

***Ec*Gyr**  ELMA.D.LEKETVDFVDNYDGTEKIPDVMPTKIPNLLVNGSSGIAVGMATNIPPHNLTEVINGCLAYIDDEDI

***Sa*TopIV** ELLR.D.INKETVSFIPNYDDTTLEPMVLPSRFPNLLVNGSTGISAGYATDIPPHNLAEVIQATLKYIDNPDI

***Sp*TopIV** YLLQ.D.IEKKTVPFAWNFDDTEKEPTVLPAAFPNLLVNGSTGISAGYATDIPPHNLAEVIDAAVYMIDHPTA

***Ec*TopIV** LLLS.E.LGQGTADWVPNFDGTLQEPKMLPARLPNILLNGTTGIAVGMATDIPPHNLREVAQAAIALIDQPKT

***Sc*TopII** KIFHPADD..PLYKYIQE.DEKTVEPEWYLPILPMILVNGAEGIGTGWSTYIPPFNPLEIIKNIRHLMNDEEL

α10’

α10’’

β10

β9

β11

β7’

***Mt*Gyr** DEEETLAAVMGRVKGPDFPTA.GLIVG...SQGTADAYKTGRGSIRMRGVVEVEE.DSRGRTSLVITELPYQV

***Ec*Gyr**  ....SIEGLMEHIPGPDFPTA.AIING...RRGIEEAYRTGRGKVYIRARAEVEVDAKTGRETIIVHEIPYQV

***Sa*TopIV** ....TVNQLMKYIKGPDFPTG.GIIQG...IDGIKKAYESGKGRIIVRSKVEEET.LRNGRKQLIITEIPYEV

***Sp*TopIV** ....KIDKLMEFLPGPDFPTG.AIIQG...RDEIKKAYETGKGRVVVRSKTEIEK.LKGGKEQIVITEIPYEI

***Ec*TopIV** ....TLDQLLDIVQGPDYPTE.AEIIT...SRAEIRKIYENGRGSVRMRAVWKKED.....GAVVISALPHQV

***Sc*TopII** ........EQ...MHPWFRGWTGTIEEIEP.............LRYRMYGRIEQIGD....NVLEITELPART

********

α11

α12

β12

β13

β14

β15

***Mt*Gyr** NHDNFITSIAEQVRDGKLAGI..SNIEDQSSDRVGLRIVIEIKRDAVAKVVI.NNLYKHTQLQTSFGA.NMLA

***Ec*Gyr**  NKARLIEKIAELVKEKRVEGI..SALRDES.DKDGMRIVIEVKRDAVGEVVL.NNLYSQTQLQVSFGI.NMVA

***Sa*TopIV** NKSSLVKRIDELRADKKVDGI..VEVRDET.DRTGLRIAIELKKDVNSESIK.NYLYKNSDLQISYNF.NMVA

***Sp*TopIV** NKANLVKKIDDVRVNNKVAGI..AEVRDES.DRDGLRIAIELKKDANTELVL.NYLFKYTDLQINYNF.NMVA

***Ec*TopIV** SGARVLEQIAAQMRNKKLPMV..DDLRDESDHENPTRLVIVPRSNRVDMDQVMNHLFATTDLEKSYRINLNMI

***Sc*TopII** WTSTIKEYLLLGLSGNDKIKPWIKDMEEQH.D.DNIKFIITLSPEEMAKTRK.IGFYERFKLISPISLMNMVA

α14

α14’

α15

β15

β16

***Mt*Gyr** IV.DGVPRTL.RLDQLIRYYVDHQLDVIVRRTTYRLRKANERAHILRGLVKALDAL..DEVIALI.RASETVD

***Ec*Gyr**  LH.HGQPKIM.NLKDIIAAFVRHRREVVTRRTIFELRKARDRAHILEALAVALANI..DPIIELI.RHAPTPA

***Sa*TopIV** IS.DGRPKLM.GIRQIIDSYLNHQIEVVANRTKFELDNAEKRMHIVEGLIKALSIL..DKVIELI.RSSKNKR

***Sp*TopIV** ID.NFTPRQV.GIVPILSSYIAHRREVILARSRFDKEKAEKRLHIVEGLIRVISIL..DEVIALI.RASENKA

***Ec*TopIV** GLDGRPAVKN.LLE.ILSEWLVFRRDTVRRRLNYRLEKVLKRLHILEGLLVAFLNI..DEVIEII.R...NED

***Sc*TopII** FDPHGKIKKYNSVNEILSEFYYVRLEYYQKRKDHMSERLQWEVEKYSFQVKFIKMIIEKELT..VTNKP..RN

α15

α15

***Mt*Gyr** IARAGLIEL..................................LDID..........................

***Ec*Gyr**  EAKTALVANPWQLGNVAAMLERAGDDAARPEWLEPEFGVRDGLYYLT..........................

***Sa*TopIV** DAKENLIEV..................................YEFT..........................

***Sp*TopIV** DAKENLKVS..................................YDFT..........................

***Ec*TopIV** EPKPALMSR..................................FGLT..........................

***Sc*TopII** AIIQELENLG.................................FPRFNKEGKPYYGSPNDEIAEQINDVKGAT

α19

α16

α18

***Mt*Gyr** .......................EIQAQAILDMQLRRLAALERQRIIDDLAKIEAEIADLEDILAKPERQRGI

***Ec*Gyr**  .......................EQQAQAILDLRLQKLTGLEHEKLLDEYKELLDQIAELLRILGSADRLMEV

***Sa*TopIV** .......................EEQAEAIVMLQLYRLTNTDIVALEGEHKELEALIKQLRHILDNHDALLNV

***Sp*TopIV** .......................EEQAEAIVTLQLYRLTNTDVVVLQEEEAELREKIAMLAAIIGDERTMYNL

***Ec*TopIV** .......................ETQAEAILELKLRHLAKLEEMKIRGEQSELEKERDQLQGILASERKMNNL

***Sc*TopII** SDEEDEESSHEDTENVINGPEELYGTYEYLLGMRIWSLTKERYQKLLKQKQEKETELENLLKL..SAKDIWNT

α19

β20

***Mt*Gyr** VRDELAEIVDRHGDDRRTRIIAA.....

***Ec*Gyr**  IREELELVREQFGDKRRTEIT.......

***Sa*TopIV** IKEELNEIKKKFKSERLSLIEAEIEE..

***Sp*TopIV** MKKELREVKKKFATPRL...........

***Ec*TopIV** LKKELQADAQAYGDDRRSPLQEREEAKA

***Sc*TopII** DLKAFEVGYQEFLQRDAEAR........

**Figure S3.** Structure-based sequence alignment of the breakage-reunion domain from type II topoisomerases. The sequence names are as follows: ***Mt*Gyr** (PDB code 3IFZ) (this work), *M. tuberculosis* DNA gyrase; ***Ec*Gyr** (PDB code 1AB4) (36)**,** *E. coli* DNA gyrase*;* ***Sa*TopIV** (PDB code 2INR) (34)**,** *S. aureus* topoisomerase IV; ***Sp*TopIV** (PDB code 2NOV) (33)**,** *S. pneumoniae* topoisomerase IV; ***Ec*TopIV** (PDB code 1ZVU) (see below)**,** *E. coli* topoisomerase IV and ***Sc*TopII** (PDB code 2RGR) (29), *S. cerevisiae* topoisomerase II. α-helices (cylinders) and β-strands (arrows) of *M. tuberculosis* GA57BK are shown with the sequences and color-coded according to Fig 1 (N-terminal helix in red, DNA-gate in blue, Tower in green, helix bundle in orange and C-gate in purple). Residues emphasized by black shading are 100 % conserved. The catalytic residues are underlined by red stars (R128 and Y129) and GA57BK specific motifs by black stars (the DPP and DEEX motifs). The QRDR-A is delimited by a blue frame.

**Reference of PDB code 1ZVU**

Corbett, K.D.,   Schoeffler, A.J.,   Thomsen, N.D.,   Berger, J.M. (2005). The structural basis for substrate specificity in DNA topoisomerase IV. J.Mol.Biol. **351:** 545-561.
