# Supplementary material for: Structural Insights into the Quinolone Resistance Mechanism of Mycobacterium tuberculosis DNA Gyrase
Source: PLoS One. 2010 Aug 18;5(8):e12245. doi: 10.1371/journal.pone.0012245 (PMC2923608; doi:10.1371/journal.pone.0012245)
Supplement: Figure S4 — Superimposition of the different monomer structures of the breakage-reunion domain. M. tuberculosis DNA gyrase GA57BK (3IFZ) (this work) in light green, M. tuberculosis DNA gyrase MtGyrA59 (3ILW, 25) in pale green, E. coli DNA gyrase (1AB4) (36) in dark green, S. pneumoniae topoisomerase IV (2NOV) (33) in red, S. aureus topoisomerase IV (2INR) (34) in pale red, S. pneumoniae complexed with DNA (3FOF) (26) in dark red and E. coli topoisomerase IV (1ZVU) in firebrick. The rmsd (in Ang.) after superimposition and the number of common Cα (in parenthesis) are indicated in the table. The color code is conserved. (0.43 MB DOC) [file pone.0012245.s005.doc]

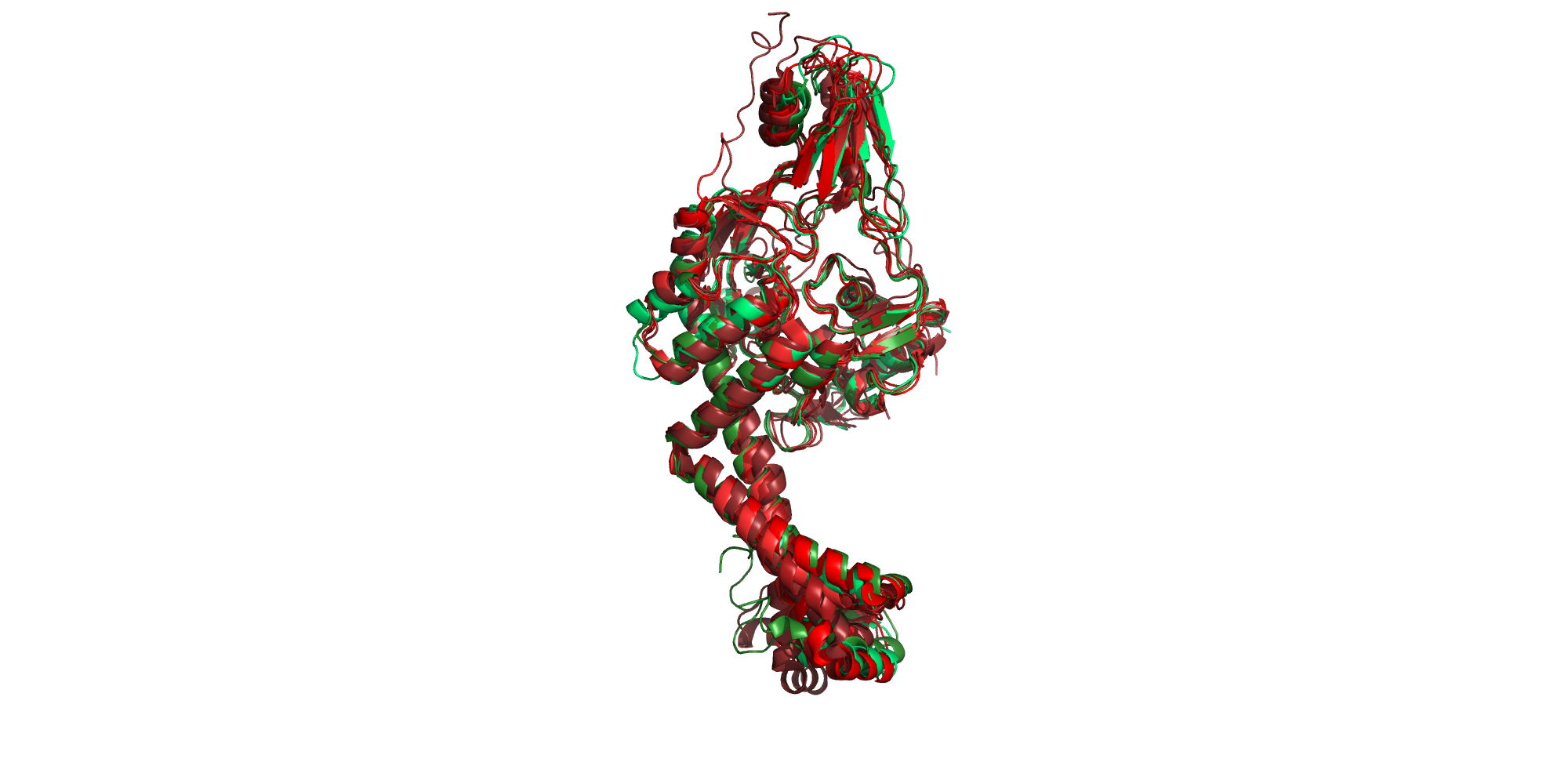


|  | **3ILW**  ***Mt*Gyr** | **1AB4**  ***Ec*Gyr** | **2NOV**  ***Sp*TopIV** | **2INR**  ***Sa*TopIV** | **3FOF**  ***Sp*TopIV** | **1ZVU**  ***Sc*TopIV** |
| --- | --- | --- | --- | --- | --- | --- |
| **3IFZ *Mt*Gyr** | 0.543 (354) | 0.847 (368) | 1.372 (399) | 0.869 (351) | 1.426 (362) | 1.576 (321) |
| **3ILW**  ***Mt*Gyr** |  | 0.991 (384) | 1.934 (396) | 0.969 (370) | 1.317 (353) | 1.796 (336) |
| **1AB4 *Ec*Gyr** |  |  | 1.813 (395) | 1.365 (387) | 1.577 (365) | 1.546 (317) |
| **2NOV *Sp*TopIV** |  |  |  | 0.904 (347) | 1.080 (339) | 1.884 (318) |
| **2INR *Sa*TopIV** |  |  |  |  | 0.867 (359) | 1.813 (326) |
| **3FOF *Sp*TopIV** |  |  |  |  |  | 2.597 (310) |

**Figure S4**. Superimposition of the different monomer structures of the breakage-reunion domain. *M. tuberculosis* DNA gyrase GA57BK (3IFZ) (this work) in light green, *M. tuberculosis* DNA gyrase *Mt*GyrA59 (3ILW, 25) in pale green, *E. coli* DNA gyrase (1AB4) (36) in dark green*, S. pneumoniae* topoisomerase IV (2NOV) (33) in red*, S. aureus* topoisomerase IV (2INR) (34) in pale red, *S. pneumoniae* complexed with DNA (3FOF) (26) in dark red and *E. coli* topoisomerase IV (1ZVU) (see below) in firebrick. The rmsd (in Å) after superimposition and the number of common C_α_ (in parenthesis) are indicated in the table. The color code is conserved.

**Additional reference**

Corbett KD, Schoeffler AJ, Thomsen ND, Berger JM (2005). The structural basis for substrate specificity in DNA topoisomerase IV. J Mol Biol. 351: 545-561.
